# Decreased Abundance of *Akkermansia muciniphila* Leads to the Impairment of Insulin Secretion and Glucose Homeostasis in Lean Type 2 Diabetes

**DOI:** 10.1002/advs.202100536

**Published:** 2021-06-04

**Authors:** Jing Zhang, Yueqiong Ni, Lingling Qian, Qichen Fang, Tingting Zheng, Mingliang Zhang, Qiongmei Gao, Ying Zhang, Jiacheng Ni, Xuhong Hou, Yuqian Bao, Petia Kovatcheva‐Datchary, Aimin Xu, Huating Li, Gianni Panagiotou, Weiping Jia

**Affiliations:** ^1^ Shanghai Key Laboratory of Diabetes Mellitus Department of Endocrinology and Metabolism Shanghai Diabetes Institute Shanghai Clinical Center for Diabetes Shanghai Jiao Tong University Affiliated Sixth People's Hospital Shanghai 200233 China; ^2^ Systems Biology and Bioinformatics Unit Leibniz Institute for Natural Product Research and Infection Biology–Hans Knöll Institute Beutenbergstrasse 11a Jena 07745 Germany; ^3^ Systems Biology & Bioinformatics Group School of Biological Sciences The University of Hong Kong Hong Kong SAR China; ^4^ Institute for Molecular Infection Biology University of Wurzburg Wurzburg D‐97080 Germany; ^5^ State Key Laboratory of Pharmaceutical Biotechnology The University of Hong Kong Hong Kong SAR China; ^6^ Department of Medicine The University of Hong Kong Hong Kong SAR China

**Keywords:** 3*β*‐chenodeoxycholic acid, *Akkermansia muciniphila*, bile acids, glucose tolerance, gut microbiota, insulin secretion, lean with type 2 diabetes

## Abstract

Although obesity occurs in most of the patients with type 2 diabetes (T2D), a fraction of patients with T2D are underweight or have normal weight. Several studies have linked the gut microbiome to obesity and T2D, but the role of gut microbiota in lean individuals with T2D having unique clinical characteristics remains unclear. A metagenomic and targeted metabolomic analysis is conducted in 182 lean and abdominally obese individuals with and without newly diagnosed T2D. The abundance of *Akkermansia muciniphila* (*A. muciniphila*) significantly decreases in lean individuals with T2D than without T2D, but not in the comparison of obese individuals with and without T2D. Its abundance correlates inversely with serum 3*β*‐chenodeoxycholic acid (*β*CDCA) levels and positively with insulin secretion and fibroblast growth factor 15/19 (FGF15/19) concentrations. The supplementation with *A. muciniphila* is sufficient to protect mice against high sucrose‐induced impairment of glucose intolerance by decreasing *β*CDCA and increasing insulin secretion and FGF15/19. Furthermore, *β*CDCA inhibits insulin secretion and *FGF15/19* expression. These findings suggest that decreased abundance of *A. muciniphila* is linked to the impairment of insulin secretion and glucose homeostasis in lean T2D, paving the way for new therapeutic options for the prevention or treatment of diabetes.

## Graphical abstract

Intestinal *Akkermansia muciniphila* is depleted in lean individuals with type 2 diabetes, who have increased 3*β*‐chenodeoxycholic acid (*β*CDCA), reduced insulin secretion and fibroblast growth factor 19 (FGF19) expression. *A. muciniphila* treatment in mice modulates *β*CDCA levels, stimulates insulin secretion and FGF19 expression, leading to improved glucose metabolism. The way is paved fornew therapeutic options for diabetes prevention or treatment.

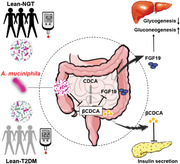

## 1 Introduction

Type 2 diabetes (T2D), a complex disease with varying manifestation and risk of complications, has become a global epidemic.^[^ ^1^ ^]^ Obesity is a main risk factor for T2D, and epidemiologic studies indicated that 70% to 80% of diabetic patients are obese.^[^ ^2^, ^3^, ^4^ ^]^ However, a fraction of patients with T2D are underweight or have normal weight.^[^ ^5^ ^]^ A study in a Scandinavian population identified distinct subgroups of individuals with T2D, representing a first step towards tailored treatment and precision medicine in diabetes.^[^ ^6^ ^]^ Compared with obese patients with T2D, lean patients with T2D have different clinical characteristics and risks of diabetic complications.^[^ ^6^, ^7^ ^]^

A growing number of studies to parse the underlying contributors to T2D have focused on gut microbiome.^[^ ^8^ ^]^ Pioneering metagenomic shotgun sequencing studies have enabled characterization of the gut microbiome in individuals with metabolic diseases,^[^ ^9^, ^10^, ^11^, ^12^ ^]^ and furthered our understanding of the functional interplay between the gut microbiota and host metabolism. Patients with T2D tend to have both compositional and functional alterations in their metagenomes compared to people without T2D.^[^ ^11^, ^12^, ^13^ ^]^ An increase in the total *Lactobacillus* and decrease in *Clostridium coccoides* group, *Atopobium* cluster, and *Prevotella* were observed in diabetic individuals.^[^ ^13^ ^]^ Accumulating evidence also suggest altered gut microbiota composition in people who are obese.^[^ ^14^ ^]^ For example, some studies showed that obese individuals contained more *Firmicutes* and fewer *Bacteroidetes*.^[^ ^15^, ^16^ ^]^ However, whether and how alterations in gut microbiota are functionally involved in the diverse clinical characteristics between lean and obese T2D remains obscure.

Alteration in host bile acids (BAs) is one way that gut microbes affect host metabolism.^[^ ^17^, ^18^, ^19^ ^]^ In humans, primary BAs are synthesized from enzymatic oxidation of cholesterol in the liver^[^ ^20^ ^]^ and these BAs are converted to secondary BAs by the metabolic activities of enteric anaerobic bacteria.^[^ ^21^ ^]^ Human fibroblast growth factor 19 (FGF19, also called FGF15 in rodents) is produced in the intestine, is a negative feedback regulator of BAs, and its expression is important for metabolic health and regulated by BAs.^[^ ^20^, ^22^ ^]^ In addition to the well‐known functions in facilitating digestion and dietary lipid absorption, BAs participate in triglyceride, energy and glucose homeostasis through binding to the nuclear receptors farnesoid X receptor (FXR) and the G‐protein‐coupled receptor 5.^[^ ^20^, ^23^ ^]^ People with T2D have altered plasma BAs composition and levels.^[^ ^24^, ^25^, ^26^ ^]^ Additionally, studies in both animals and humans show that BA treatment improves glycemic control.^[^ ^27^, ^28^ ^]^

Here we compared the gut microbiome of lean and abdominally obese participants with and without T2D, to determine if differences in gut microbiota could explain the specific clinical characteristics of lean individuals with T2D. By combining metagenomics and targeted metabolomics, we found that lean patients with T2D had distinct gut microbiota and BA profiles. We also identified possible effects of specific components of the gut microbiota on host BA profiles and metabolism. The roles of identified intestinal microbial species and metabolite were validated using both in vivo mouse model and in vitro cell lines. These results suggest the involvement of gut microbiota alterations in the pathogenesis of lean T2D and shed light on potential microbiome‐focused strategies for the prevention or treatment of T2D in lean individuals.

## 2 Results

### 2.1 Participant Characteristics

**Figure 1:**
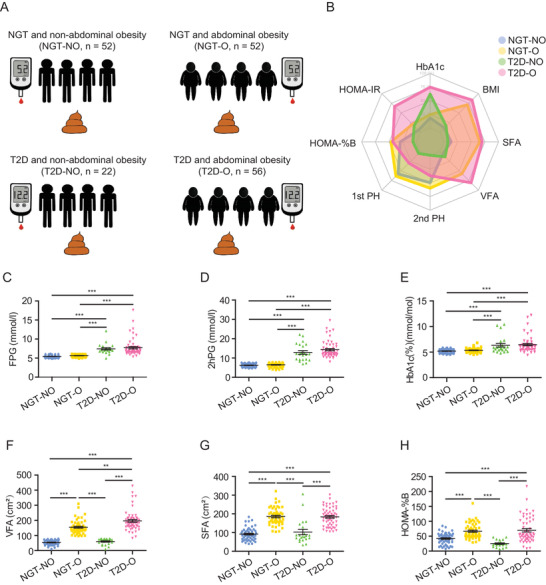
Overall study design and major clinical data among the four groups. A) Study design. B) Radar plot of four groups showing overall different clinical characteristics related to T2D and abdominal obesity. C–H) Boxplots with major clinical outcomes for participants in the four groups. Data are means ± SEM. Statistical significance was determined with ANOVA with Bonferroni multiple‐comparison analysis. BMI, body mass index; FPG, fasting plasma glucose; HOMA‐%B, homeostasis model assessment of insulin secretion; HOMA‐IR, homeostasis model assessment of insulin resistance; 2hPG, 2‐h plasma glucose during OGTT; NGT‐NO, normal glucose tolerance‐lean; NGT‐O, normal glucose tolerance‐abdominally obese; SFA, subcutaneous fat area; T2D‐NO, type 2 diabetes‐lean; T2D‐O, type 2 diabetes‐abdominally obese; VFA, visceral fat area. 1st PH, first‐phase insulin release; 2nd PH, second‐phase insulin release. ^**^ *p* < 0.01; ^***^ *p* < 0.001.

Four groups of gender‐matched adult participants were selected (see the Experimental Section for detailed inclusion and exclusion criteria) from the Shanghai Nicheng cohort study^[^ ^29^ ^]^ from May 2014 to June 2014, including the NGT‐lean (normal glucose tolerance‐lean, NGT‐NO) group (*n* = 52), NGT‐abdominally obese (NGT‐O) group (*n* = 52), lean with newly diagnosed T2D (T2D‐NO) group (*n* = 22), and abdominally obese with newly diagnosed T2D (T2D‐O) group (*n* = 56) (**Figure** **1**A). Clinical characteristics of the study participants are shown in **Table** **1**. No significant differences in body mass index (BMI), waist circumference, thigh circumference, visceral fat area (VFA), and subcutaneous fat area (SFA) were observed between the T2D‐NO group and the NGT‐NO group. No significant differences in fasting plasma glucose (FPG), 2 h plasma glucose during oral glucose tolerance test (OGTT) (2hPG), and HbA1c were observed between the T2D‐NO group and the T2D‐O group. Among the four groups, participants in T2D‐NO had the lowest homeostasis model assessment of insulin secretion (HOMA‐% B), first‐phase insulin release (1st PH) and second‐phase insulin release (2nd PH), suggesting dysfunction of insulin secretion (Figure 1B–H). We found that fasting FGF19 levels in participants in the T2D‐NO and T2D‐O groups were significantly lower than in the NGT‐NO group (*p* < 0.05) (Table 1). No significant differences of educational level, alcohol intake, and smoking habits were found among the four groups (Table 1).

**Table 1:** Anthropometric parameters and biochemical indexes among participants (*n* = 182)

Variables	NGT‐NO (*n* = 52)	NGT‐O (*n* = 52)	T2D‐NO (*n* = 22)	T2D‐O (*n* = 56)	*p*
Male/female	29/23	26/26	11/11	30/26	–
Age (year)	54.3 ± 6.8	56.0 ± 5.4	59.0 ± 5.3*	58.1 ± 6.8*	0.004
BMI (kg m^−2^)	21.3 ± 1.6	27.6 ± 1.9^*†^	22.4 ± 3.9	28.9 ± 2.9^*†‡^	<0.001
Waist circumference (cm)	71.5 ± 5.5	91.9 ± 5.0^*†^	74.3 ± 8.6	94.8 ± 6.7^*†^	<0.001
Thigh circumference (cm)	47.6 ± 3.3	53.2 ± 3.8^*†^	47.6 ± 4.1	52.4 ± 3.4^*†^	<0.001
FPG (mmol L^−1^)^§^	5.5 (5.2, 5.6)	5.7 (5.4, 6.0)^†^	7.2 (6.8, 7.8)*	7.1 (6.6, 7.8)^*‡^	<0.001
2hPG (mmol L^−1^)^§^	6.2 (5.4, 7.0)	6.8 (5.7, 7.3)^†^	12.0 (9.1, 17.0)*	12.5 (11.8, 15.7)^*‡^	<0.001
VFA (cm^2^)^§^	53.5 (41.2, 67.5)	141.9 (125.5, 174.3)^*†^	66.9 (44.0, 75.6)	183.3 (158.2, 215.0)^*†‡^	<0.001
SFA (cm^2^)^§^	89.0 (66.3, 109.9)	179.3 (154.8, 209.2)^*†^	80.9 (65.1, 132.9)	185.5 (138.1, 219.4)^*†^	< 0.001
VFA/SFA	0.6 (0.5, 0.8)	0.9 (0.6, 1.1)*	0.6 (0.5, 1.0)	1.0 (0.8, 1.4)^*†‡^	<0.001
HbA1c (%)^§^	5.2 (4.9, 5.5)	5.3 (5.1, 5.6)^†^	5.8 (5.4, 6.5)*	6.1 (5.5, 6.5)^*‡^	<0.001
FINS (µU mL^−1^)^§^	4.0 (2.8, 5.5)	6.1 (5.2, 8.4)*	4.7 (2.7, 7.6)	11.1 (8.0, 15.3)^*†‡^	<0.001
2hINS (µU mL^−1^)^§^	25.9 (16.4, 33.5)	33.4 (19.9, 43.1)	42.3 (23.2, 50.8)	80.6 (48.8, 112.4)^*†‡^	<0.001
HOMA‐IR^§^	0.9 (0.6, 1.4)	1.5 (1.3, 2.1)*	1.5 (0.9, 2.5)*	3.7 (2.5, 5.4)^*†‡^	< 0.001
HOMA‐%B^§^	43.8 (29.1, 55.7)	57.7 (49.7, 82.5)^*†^	24.1 (18.8, 37.9)	55.1 (40.9, 101.6)^*†^	<0.001
1st PH	713.0 ± 58.5	985.2 ± 87.1^†^	85.5 ± 199.9*	512.3 ± 112.3^‡^	< 0.001
2nd PH	208.4 ± 13.0	275.8 ± 19.3^†^	89.8 ±45.4*	188.8 ± 23.9^‡^	<0.001
FGF19 (pg mL^−1^)^§^	278.8 (136.6, 581.3)	207.0 (122.7, 346.6)	165.9 (78.2, 324.4)*	213.3 (112.2, 391.1)*	0.095
Educational level (*a*/*b*/*c*/*d*/*e*)	2/27/18/5/0	5/23/20/4/0	2/12/5/3/0	9/24/17/5/1	0.422
No drinking/drinking	44/8	45/7	19/3	41/15	0.105
No smoking/smoking	33/19	38/14	19/3	41/15	0.217
Prevalence of fatty liver	5.8%	76.5%^*†^	23.8%	85.7%^*†^	<0.001
Exercise intensity (*a*/*b*/*c*)	52/0/0	50/1/1	20/0/2	52/2/2	0.072

Data are means ± SD or median (interquartile range). Data are means ± SEM for 1st PH or 2nd PH. Statistical significance was determined with ANOVA with Bonferroni multiple‐comparison analysis. § Log transformed before analysis. BMI, body mass index; FINS, fasting serum insulin; FGF19, Fibroblast growth factor 19; FPG, fasting plasma glucose; HOMA‐%B, homeostasis model assessment of insulin secretion; HOMA‐IR, homeostasis model assessment of insulin resistance; 2hPG, 2‐h plasma glucose during OGTT; 2hINS, 2 h serum insulin during OGTT; NGT‐NO, normal glucose tolerance‐lean; NGT‐O, normal glucose tolerance‐abdominally obese; SFA, subcutaneous fat area; T2D‐NO, type 2 diabetes‐lean; T2D‐O, type 2 diabetes‐abdominally obese; VFA, visceral fat area; 1st PH, first‐phase insulin release; 2nd PH, second‐phase insulin release; educational level (*a*/*b*/*c*/*d*/*e*), no education/primary education/lower secondary education/upper secondary or intermediate vocational education/higher vocational education; exercise intensity (*a*/*b*/*c*), no exercise/less than half an hour of exercise every day/at least half an hour of exercise every day. * versus NGT‐NO, *p* < 0.05; † versus T2D‐NO, *p* < 0.05; ‡, versus NGT‐O, *p* < 0.05.

### 2.2 Different Gut Microbiome Compositions in the Four Groups

Whole‐genome shotgun metagenomic sequencing was performed on fecal samples of all 182 participants, generating an average of 31.2 million high‐quality reads for each sample. In our cohort of healthy people and those with newly diagnosed T2D and/or abdominal obesity, *Bacteroides*, *Firmicutes*, and *Proteobacteria* were the most dominant phyla across all groups (Figure S1A, Supporting Information). These phyla accounted for more than 96% of the gut microbial composition, with other bacteria present at much lower abundance (less than 4% on average) (Figure S1B, Supporting Information). The T2D‐NO group on average had a higher relative abundance of phylum *Firmicutes* (30%) and lower abundance of phylum *Bacteroidetes* (62%) than the other three groups. However, no significant differences were observed at the phylum level among the four groups (e.g., *p* = 0.17 for *Firmicutes*, *p* = 0.31 for *Bacteroidetes*, Kruskal–Wallis test).

**Figure 2:**
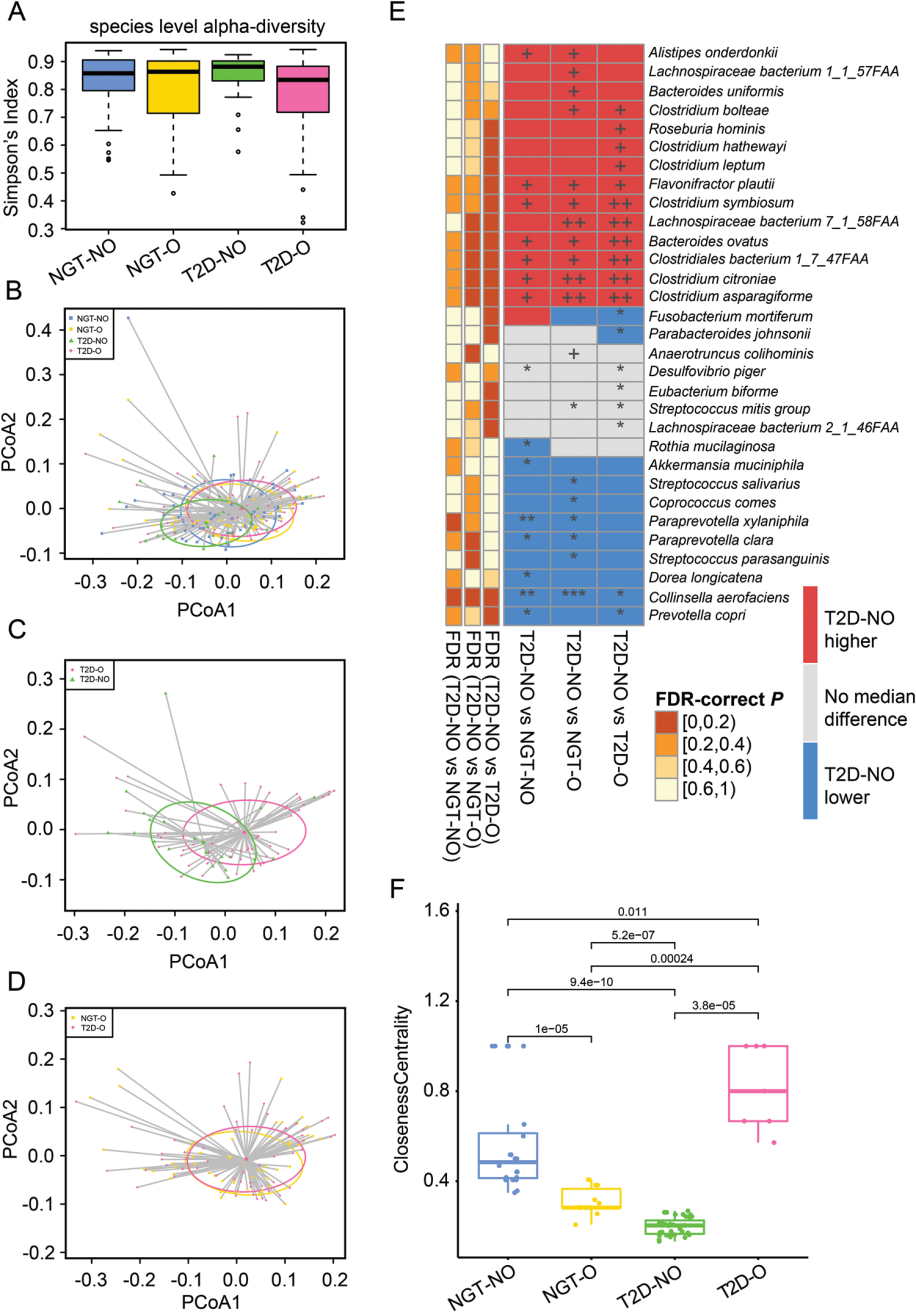
Comparison of the microbial community diversity and composition. A) Microbial alpha‐diversity (Simpson index) in different groups. PCoA plots based on weighted UniFrac distances comparing B) four groups; C) T2D‐NO versus T2D‐O; D) NGT‐O versus T2D‐O. E) Differential abundance analysis to assess significant elevation or depletion in T2D‐NO compared with each of the other three groups. Red/blue: higher/lower median abundance in T2D‐NO. Gray, no difference in median abundance. ^*/+^ *p* < 0.05; ^**/++^ *p* < 0.01; ^***/+++^ *p* < 0.001 (one‐sided Wilcoxon rank‐sum test, * for “less” and + for “greater”). F) Comparison of node closeness centrality for co‐abundant networks in different groups. The horizontal lines above indicate comparisons between two groups on the ends (Wilcoxon rank‐sum test). In (A,F) boxplots show median (centerlines), lower/upper quartiles (box limits), whiskers (the last data points 1.5 times interquartile range (IQR) from the lower or upper quartiles), and notches (95% confidence interval for the medians). PCoA, principal coordinate analysis; NGT‐NO, normal glucose tolerance‐lean; NGT‐O, normal glucose tolerance‐abdominally obese; T2D‐NO, type 2 diabetes‐lean; T2D‐O, type 2 diabetes‐abdominally obese; PCoA, principal coordinate analysis.

After taxonomic profiling, we calculated and compared community‐level microbiota diversity among the four groups. No significant differences in alpha diversity (Shannon and Simpson) were observed, with the exception of the comparison between the two groups with diabetes (T2D‐NO vs T2D‐O) (*p* < 0.05, Wilcoxon rank‐sum test) (**Figure** **2**A and Table S1, Supporting Information). The T2D‐NO group had higher alpha diversity than the T2D‐O group. While principal coordinate analysis (PCoA) with phylogenetic‐based unweighted UniFrac distance did not show a clear separation among the four groups (Figure S1C, Supporting Information), the distinct microbial composition of the T2D‐NO group was captured by weighted UniFrac distance that considers the species abundances when calculating beta diversity (Figure 2B). This finding suggested that despite highly similar sets of bacteria in all four groups, the abundance distribution might be unique in the T2D‐NO group compared with the other three groups. More specifically, significant differences were seen between the T2D‐NO and T2D‐O groups (*p* = 0.012, PERMANOVA) (Figure 2C), and the T2D‐NO and NGT‐O groups (*p* = 0.03, PERMANOVA) (Table S1, Supporting Information). In spite of a trend of separation between the NGT‐NO and T2D‐NO groups, no significant difference was found at the community level (*p* = 0.108, PERMANOVA). Interestingly, we found from the PCoA that the NGT‐O and T2D‐O groups shared highly similar overall gut microbial community profiles (Figure 2D) (*p* = 0.918, PERMANOVA).

Next, we performed pairwise differential abundant species analysis to identify specific bacteria with potential clinical importance, focusing on the T2D‐NO group that had unique clinical characteristics (e.g., decreased insulin secretion) and microbiota composition. Following the identification of significant differences between the T2D‐NO and NGT‐O groups and T2D‐NO and T2D‐O groups based on microbial beta diversity, we found 27 species significantly differentially abundant in both the T2D‐NO versus NGT‐O and T2D‐NO versus T2D‐O comparisons, with several species in common between the two comparisons (Figure 2E) (*p* < 0.05, Wilcoxon rank‐sum test). Although the T2D‐NO and NGT‐NO control groups did not show significant community‐level differences, individual species were found differentially abundant between the two groups (Figure 2E), e.g., *Akkermansia muciniphila* (*A. muciniphila*), *Paraprevotella xylaniphila*, *Ruminococcaceae bacterium d16*, and *Prevotella copri*, suggesting that T2D‐NO and NGT‐NO groups were characterized by certain microbes rather than by large variation in the entire community.

We then constructed microbial co‐abundance networks and investigated the species interactions in the four different metabolic conditions. While the T2D‐NO group network seemed to be similar in size to the NGT‐NO group network (Figure S2A, Supporting Information), it was not well connected, with only one key “bridge” interaction between *Faecalibacterium prausnitzii* and *Bacteroides stercoris*. To support our conjecture, we determined several commonly used network topological properties such as average shortest path length, closeness centrality and clustering coefficient (Figure 2F and Figure S2B, Supporting Information). Closeness centrality was significantly lower in the T2D‐NO group than the NGT‐NO group (*p* < 0.05, Wilcoxon rank‐sum test). The bacterial co‐abundance network in T2D‐O group was quite small and uninformative, especially compared with the other three groups (Figure S2A, Supporting Information). The NGT‐O network was relatively smaller and simpler than the NGT‐NO control group but had more complexity than the T2D‐O group (Figure 2F and Figure S2A‐B, Supporting Information).

### 2.3 Functional Characterization of the Microbiome

Next, we performed Kyoto encyclopedia of genes and genomes (KEGG) functional module comparisons to uncover potential critical microbiome functions within the four groups. The four groups exhibited notable divergent module enrichment (Figure S3A, Supporting Information). For example, the gut microbiota of T2D patients (T2D‐NO and T2D‐O groups) showed enrichment in “pantothenate and CoA biosynthesis,” “flavone and flavonol biosynthesis,” “amino sugar and nucleotide sugar metabolism,” and “tyrosine metabolism.” The gut microbiota of patients with abdominal obesity (NGT‐O and T2D‐O groups) showed enrichment in “ubiquinone and other terpenoid‐quinone biosynthesis” and “tropane, piperidine and pyridine alkaloid biosynthesis.” We then investigated differential microbial functions between the T2D‐NO and NGT‐NO groups, seeking insights into the potential pathological effects of gut microbiota on the T2D‐NO phenotype (Figure S3B, Supporting Information). T2D‐NO individuals were depleted in the functional potential for phosphotransferase systems involved in the uptake and phosphorylation of a variety of carbohydrates.^[^ ^30^ ^]^ The “valine, isoleucine biosynthesis” functional module was increased in the T2D‐NO microbiome. Of the three branched‐chain amino acids (BCAAs), circulating valine in subjects with T2D‐NO were significantly higher than that in NGT‐NO subjects (T2D‐NO 178.8 ± 28.4 × 10^−6^ m vs NGT‐NO 151.1 ± 24.7 × 10^−6^ m, *p* = 0.013). The levels of leucine (T2D‐NO 109.2 ± 19.6 × 10^−6^ m vs NGT‐NO 96.3 ± 15.0 × 10^−6^ m, *p* = 0.061) and isoleucine (T2D‐NO 48.8 ± 9.6 × 10^−6^ m vs NGT‐NO 42.2 ± 7.4 × 10^−6^ m, *p* = 0.09) in subjects with T2D‐NO also had the tendency to be higher than those in NGT‐NO subjects (Table S3, Supporting Information). The correlation between elevated BCAAs and later risk for diabetes has been previously reported.^[^ ^31^, ^32^ ^]^ Our data demonstrated that the microbiota of T2D‐NO individuals may have a lower capacity for carbohydrate utilization and a higher capacity for production of BCAAs. In addition, KEGG modules involved in carbohydrate metabolism including “galactose degradation,” were highly enriched in the T2D‐NO compared to the NGT‐NO microbiome.

### 2.4 *A. muciniphila* Abundance Was Decreased in T2D‐NO and Positively Correlated with Insulin Secretion

**Figure 3:**
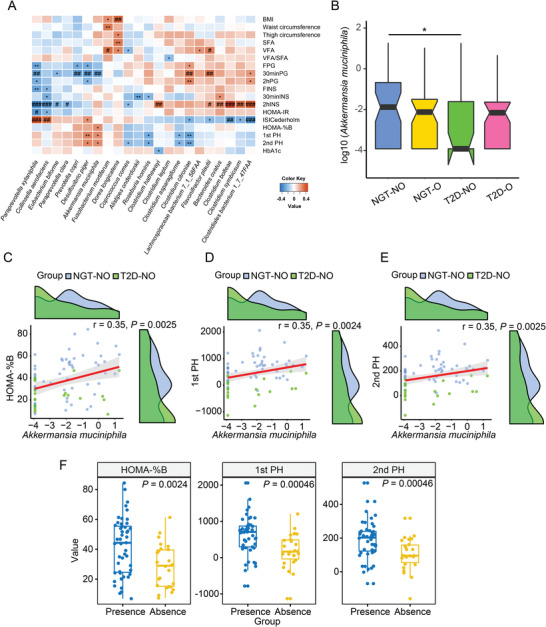
*A. muciniphila* positively related with insulin secretion. A) Partial spearman's correlations between prevalent species (>30% in at least one group) and clinical metadata after adjusting for age using T2D‐NO and NGT‐NO groups. Red, positive correlations; blue, negative correlations. FDR‐corrected ^#^ *p* < 0.1; FDR‐corrected ^##^ *p* <0.05; FDR‐corrected ^###^ *p* < 0.01; *p < 0.05; ^**^ *p* < 0.01; ^***^ *p* < 0.001. B) Metaphlan2‐derived log10‐transformed relative abundance of *A. muciniphila* by group. *p* Values from Wilcoxon rank‐sum test, *p < 0.05. C–E) Scatter plots showing the correlations between *A. muciniphila* abundance in the gut and insulin secretion. The clinical metadata related to insulin secretion used here include C) HOMA‐%B; D) 1st PH; E) 2nd PH (see the Experimental Section for their calculation). Spearman's rank correlations were used. Density distribution for *A. muciniphila* abundance and the insulin secretion‐related indexes are also plotted on the corresponding axes. For visualization purpose, the relative abundance of *A. muciniphila* is additively smoothed by half the smallest non‐zero abundance and then log10‐transformed. Blue dots, NGT‐NO samples; green dots, T2D‐NO samples. F) Comparison of insulin secretion indexes according to the presence and absence of *A. muciniphila* using T2D‐NO and NGT‐NO groups. Statistical significance was assessed with Wilcoxon rank‐sum test. In (B,F) boxplots show median (centerlines), lower/upper quartiles (box limits), whiskers (the last data points 1.5 times IQR from the lower or upper quartiles), and notches (95% confidence interval for the medians). *A. muciniphila, Akkermansia muciniphila*; BMI, body mass index; FINS, fasting serum insulin; FPG, fasting plasma glucose; HOMA‐%B, homeostasis model assessment of insulin secretion; HOMA‐IR, homeostasis model assessment of insulin resistance; 30 min PG, 30 min plasma glucose during OGTT; 30 min INS, 30 min serum insulin during OGTT; 2hPG, 2 h plasma glucose during OGTT; 2hINS, 2 h serum insulin during OGTT; NGT‐NO, normal glucose tolerance‐lean; NGT‐O, normal glucose tolerance‐abdominally obese; SFA, subcutaneous abdominal fat area; T2D‐NO, type 2 diabetes‐lean; T2D‐O, type 2 diabetes‐abdominally obese; VFA, visceral abdominal fat area. 1st PH, first‐phase insulin release; 2nd PH, second‐phase insulin release.

The four groups had different clinical profiles for body weight‐related measures, insulin sensitivity, and insulin secretion. Therefore, to prioritize marker species linking microbiota and metabolic status, we examined possible correlations between the abundances of microbial species and clinically important metadata. To investigate whether gut microbiota members are associated with the unique clinical characteristics of T2D‐NO, we determined Spearman's correlations between prevalent species (>30% in at least one group) and clinical features using all participants in the T2D‐NO and NGT‐NO control groups. The close relationship of gut microbial species with body weight‐related measures and glucose metabolism was observed before (Figure S4A, Supporting Information) and after adjusting for age (**Figure** **3**A). The bacteria *A. muciniphila*, previously reported as a probiotic that can relieve obesity,^[^ ^33^ ^]^ was significantly lower in the T2D‐NO than the NGT‐NO group (Figure 3B, *p* < 0.05, Wilcoxon rank‐sum test), but not significantly different between T2D‐O and NGT‐O group. Moreover, *A. muciniphila* positively correlated with all insulin secretion‐related indexes measured here (HOMA‐%B, 1st PH, 2nd PH) (Figure 3C–E), which still remained significant after adjusting for age (Figure 3A). In addition, the three insulin secretion measures were significantly higher in the samples with *A. muciniphila* present than those absent of *A. muciniphila* (Figure 3F), further supporting the potential association between *A. muciniphila* in the gut and insulin secretion. This positive correlation between *A. muciniphila* and insulin secretion was not observed in correlation analysis of other groups (Figure S4B–D, Supporting Information). No significant associations between *A. muciniphila* and insulin sensitivity (homeostasis model assessment of insulin resistance (HOMA‐IR) and Cederholm index (ISICederholm)) were found (Figure 3A).

### 2.5 The Relationship between Plasma BAs with Clinical Parameters and *A. muciniphila*

**Figure 4:**
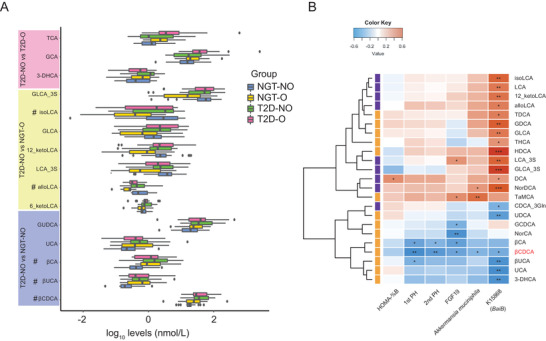
Relationship between *A. muciniphila* with FGF19 and bile acids. A) Comparison of serum BAs measured by targeted metabolomics. Only BAs that were significantly different between T2D‐NO and any of the other three groups are shown (*p* < 0.05, Student's *t*‐test based on log‐transformed data); FDR‐corrected ^#^ *p* < 0.1. Boxplots show median (centerlines), lower/upper quartiles (box limits), whiskers (the last data points 1.5 times IQR from the lower or upper quartiles), and notches (95% confidence interval for the medians). B) Correlations among measured BAs and insulin secretion‐related metadata, FGF19 levels, *A. muciniphila*, and gene K15868 for T2D‐NO and NGT‐NO groups. Only BAs with at least one significant correlation are displayed. BAs marked with purple vertical bar had higher average abundance in NGT‐NO; BAs with orange were higher in T2D‐NO. K15868: key gene (*baiB*) in secondary bile acid biosynthesis pathway (KEGG pathway ko00121). *p < 0.05; ^**^ *p* < 0.01; ^***^ *p* < 0.001, Spearman's rank correlation. Red, positive correlations; blue, negative correlations; *A. muciniphila*, *Akkermansia muciniphila*; FGF19, fibroblast growth factor 19; HOMA‐%B, homeostasis model assessment of insulin secretion; NGT‐NO, normal glucose tolerance‐lean; NGT‐O, normal glucose tolerance‐abdominally obese; SFA, subcutaneous abdominal fat area; T2D‐NO, type 2 diabetes‐lean; T2D‐O, type 2 diabetes‐abdominally obese; 1st PH, first‐phase insulin release; 2nd PH, second‐phase insulin release; *β*‐UCA, *β*‐ursocholic acid; UCA, ursocholic acid; GUDCA, glycoursodeoxycholic acid; GCA, glycocholic acid; T*α*MCA, tauro *α*‐muricholic acid; THCA, taurohyocholic acid; TCA, taurocholic acid; alloLCA, allolithocholic acid; isoLCA, isolithocholic acid; LCA, lithocholic acid; NorDCA, 23‐nordeoxycholic acid; 12‐ketoLCA, 12‐ketolithocholic acid; DCA, deoxycholic acid; GLCA, glycolithocholic acid; LCA‐3S, lithocholic acid‐3‐sulfate; 6‐ketoLCA, 6‐ketolithocholic acid; *β*CDCA, 3*β*‐chenodeoxycholic acid; UDCA, ursodeoxycholic acid; HDCA, *α*‐hyodeoxycholic acid; NorCA, norcholic acid; 3‐DHCA, 3‐dehydrocholic acid; *β*CA, 3*β*‐cholic acid; GCDCA, glycochenodeoxycholic acid; GDCA, glycodeoxycholic acid; TDCA, taurodeoxycholic acid; GLCA‐3S, glycolithocholic acid‐3‐sulfate; CDCA‐3Gln, chenodeoxycholic acid‐3‐*β*‐d‐glucuronide.

Perturbations in the gut microbiome could affect the BA pool size and composition, and alterations in plasma BAs are linked to metabolic disorders.^[^ ^20^, ^34^ ^]^ Thus, we investigated whether BAs were involved in the process of *A. muciniphila* affecting insulin secretion. We selected 20 participants per group, matched for sex and age, as a subgroup for targeted metabolomic profiling of serum BAs (Table S2, Supporting Information). No significant differences were found in the main clinical characteristics between the subgroup and the total cohort, such as VFA, SFA, FPG, and 2hPG (Table S2, Supporting Information). Of 37 identified serum BAs, a total of 20 were significantly different among the four groups (Table S3, Supporting Information). Of these 20, we identified 5 BAs with different levels between the T2D‐NO and NGT‐NO groups: glycoursodeoxycholic acid, 3*β*‐chenodeoxycholic acid (*β*CDCA), *β*‐ursocholic acid, 3*β*‐cholic acid, and urocanic acid (**Figure** **4**A).

We then determined Spearman's correlations among clinical parameters, BAs and the gut microbiome for the NGT‐NO and T2D‐NO groups. We found that *β*CDCA negatively correlated with the relative abundance of *A. muciniphila* and the insulin secretion indices 1st PH and 2nd PH (Figure 4B). Metagenomic functions were further incorporated into the correlation analysis to explore how *A. muciniphila* influenced BA metabolism. Notably, the metagenomic abundance of a key gene for bile acid‐coenzyme A ligase (BA‐induced operon, *baiB*) (KEGG Ortholog K15868) was positively associated with *A. muciniphila* abundance (Figure S5, Supporting Information) and was lower in T2D‐NO than NGT‐NO group (*p* = 0.06, Wilcoxon rank‐sum test). *baiB* is involved in the transformation of the primary BA chenodeoxycholic acid (CDCA) to chenodeoxycholoyl‐CoA that is further used for downstream secondary BA biosynthesis. Furthermore, *baiB* abundance was negatively associated with *β*CDCA (Figure 4B), the product of alternative CDCA‐utilizing route within the secondary BA biosynthesis pathway (KEGG ko00121). Taken together, the results showed that the T2D‐NO group had lower abundance of *A. muciniphila* and *baiB*, decreased insulin secretion, as well as higher *β*CDCA, implied more transformation of CDCA into *β*CDCA, suggesting that the link between *A. muciniphila* and insulin secretion might be mediated by the *β*CDCA level.

### 2.6 Effects of *A. muciniphila* on Insulin Secretion

**Figure 5:**
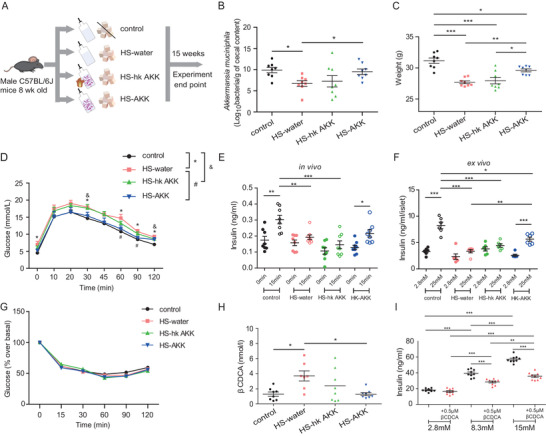
Effects of *A. muciniphila* on host metabolism. A) Schematic diagram of *A. muciniphila* supplementation study design. HS‐induced C57BL/6J mice were given drinking water (HS‐water) or water with heat‐killed (HS‐hk AKK) or live *A. muciniphila* (HS‐AKK) for 15 weeks. B) Abundance of *A. muciniphila* quantified by qPCR with specific primers to determine cecum content for groups. C) Body weight, D) glucose tolerance test, E) in vivo GSIS for mice by group. F) Glucose stimulated insulin secretion in islets isolated from the four groups. Insulin concentration was measured at 60 min after stimulation. G) Insulin sensitivity in the four groups of mice determined by ITT. H) *β*CDCA levels in mouse serum determined for the four groups. I) Treatment of MIN6 cell line with *β*CDCA led to decreased insulin secretion. MIN6 cells were stimulated for 1 h with 2.8, 8.3, and 15 mmol L^−1^ glucose in the presence or absence of 0.5 µmol L^−1^ *β*CDCA. Data are presented as mean ± SEM for figures with error bars. *N* = 7–8 mice per group for (B–E,G–H). *N* = 6 mice per group for (F). Three independent experiments were performed. Significance was determined by Student's *t*‐test analysis E) 0 min versus 15 min per group; F) 2.8 × 10^−3^ m versus 25 × 10^−3^ m per group; I) with 0.5 × 10^−6^ m *β*CDCA versus without 0.5 × 10^−6^ m *β*CDCA per concentration) and one‐way ANOVA with C,E–I) Bonferroni or B,D) Fisher's LSD multiple‐comparison analysis for comparing of more than two groups. *, #, or & *p* < 0.05; ^**^ *p* < 0.01; ^***^ *p* < 0.001.

The above analysis indicated that the relative abundance of *A. muciniphila* was lower in lean participants with T2D compared to those with NGT‐NO, and that the abundance of *A. muciniphila* was positively associated with insulin secretion as assessed by HOMA‐% B, 1st PH and 2nd PH. On this basis, we next examined the possible impact of *A. muciniphila* on host metabolism. We used a high‐sucrose (HS) diet to model T2D‐NO in mice.^[^ ^35^, ^36^ ^]^ Eight‐week male C57BL/6J mice fed the HS diet were treated with drinking water (HS‐water) or water with viable (HS‐AKK) or heat‐killed *A. muciniphila* (HS‐hk AKK) for 15 weeks. Mice in the control group were fed a normal chow diet and autoclaved water (**Figure** **5**A). Real‐time polymerase chain reaction (RT‐PCR) showed a significantly reduced amount of *A. muciniphila* in the feces of HS diet‐fed mice compared with mice fed a normal chow diet. Treatment with 5 × 10^10^ colony‐forming units (CFU) mL^−1^ live *A. muciniphila* for 15 weeks was sufficient to restore the level diminished by the HS diet (Figure 5B). After 15 weeks, the mice fed with HS diet (HS‐water group) had lower body weight than that of the control group (Figure 5C). Meanwhile, the HS diet also resulted in impaired glucose tolerance and blunted insulin secretion upon glucose stimulation (Figure 5D,E). The body weight of the mice in the HS‐AKK group was significantly higher than the mice in the HS‐water group. However, the elevation in body weight was not found in HS‐hk AKK group (Figure 5C). Treatment with viable instead of heat‐killed *A. muciniphila* substantially improved glucose tolerance (Figure 5D). Furthermore, we investigated the effect of *A. muciniphila* on insulin secretion both in vivo and ex vivo (Figure 5E,F). In vivo, no significant difference in fasting insulin secretion was observed among the four groups. Glucose‐stimulated insulin secretion was restored after *A. muciniphila* intervention. However, the improvement of insulin secretion did not occur in HS‐hk AKK group (Figure 5E). In our ex vivo experiment, islets were isolated from these four groups of mice. Glucose concentration of 25 mmol L^−1^ led to a robust increase in insulin release versus 2.8 mmol L^−1^ in the control group, while the islets isolated from HS‐water group showed an impaired insulin release. In agreement with the in vivo finding, the islets isolated from HS‐AKK group showed improved glucose‐stimulated insulin secretion, which was absent in the islets isolated from HS‐hk AKK group (Figure 5F). No significant difference was observed in insulin sensitivity among the four groups (Figure 5G). Taken together, these results demonstrate that the mice model with obvious impaired insulin secretion but not insulin resistance exhibits reduced amount of *A. muciniphila* and replenishment with viable *A. muciniphila* restores insulin secretion and improves glucose tolerance.

### 2.7 *A. muciniphila* Treatment Reduces *β*CDCA Levels and Stimulates Insulin Secretion

We further investigated the influence of *A. muciniphila* on serum BAs using mice. Metabolomics profiling of 27 BAs showed that the levels of five BAs were significantly different among the four mice groups (Table S4, Supporting Information): hyocholic acid, *α*‐hyodeoxycholic acid, *ω*‐muricholic acid, lithocholic acid, and *β*CDCA. Circulating *β*CDCA levels were significantly higher in HS‐water group compared to control group, and treatment with live *A. muciniphila* substantially reduced *β*CDCA levels. However, administration of heat‐killed *A. muciniphila* did not result in significant decreases in *β*CDCA levels (Figure 5H). These results were consistent with the negative correlation between *A. muciniphila* abundance and *β*CDCA levels observed in our human cohort.

A previous study reported that the BAs taurochenodeoxycholate and glycine‐conjugated chenodeoxycholate increase insulin secretion.^[^ ^37^ ^]^ As we found a negative correlation between *β*CDCA and insulin secretion, we further examined possible roles of *β*CDCA on insulin secretion. The mouse pancreatic beta cell line MIN6 was cultured with various concentrations of glucose in the presence or absence of *β*CDCA. Stimulation of MIN6 cells with glucose resulted in a significant induction of insulin levels. Treatment of MIN6 cells with glucose together with *β*CDCA inhibited the glucose‐induced insulin secretion (Figure 5I). The above results suggested that *A. muciniphila* might stimulate insulin secretion by reducing the levels of *β*CDCA in the gut.

### 2.8 *A. muciniphila* Enhances FGF15/19 through Inhibition of *β*CDCA

**Figure 6:**
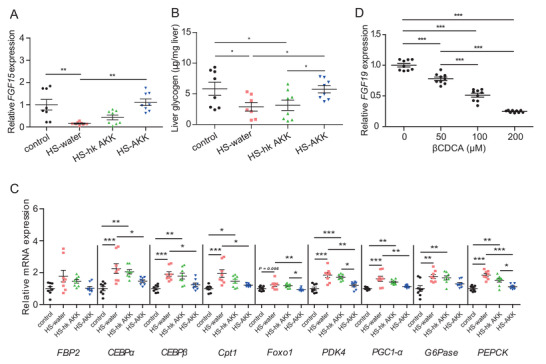
Effects of *A. muciniphila* on FGF15/19 expression. A) FGF15 mRNA levels in mouse small intestine. B) Liver glycogen content for mice by group. C) Expression of genes involved in gluconeogenesis in mouse liver. D) Treatment of LS174T cells with *β*CDCA led to decreased FGF19 expression. LS174T cells were stimulated for 24 h with 0, 50, 100, and 200 µmol L^−1^ *β*CDCA in the presence of 150 µmol L^−1^ CDCA. Data are presented as mean ± SEM for figures with error bars. Three independent experiments were performed. A–C) *N* = 7–8 mice per group. Significance was determined by one‐way ANOVA with A,C,D) Bonferroni post hoc test or B) Fisher's LSD post hoc test. *p < 0.05; ^**^ *p* < 0.01; ^***^ *p* < 0.001. HS‐induced C57BL/6J mice were given drinking water (HS‐water) or water with heat‐killed (HS‐hk AKK), or live *A. muciniphila* (HS‐AKK) for 15 weeks.

FGF19 has been shown to stimulate glycogen synthesis and inhibit gluconeogenesis through insulin‐independent pathways.^[^ ^38^ ^]^ Our clinical analysis showed that serum FGF19 levels were significantly decreased in the T2D‐NO group compared to the NGT‐NO group. Moreover, the FGF19 level was positively correlated with the abundance of *A. muciniphila* in the gut microbiota and negatively associated with *β*CDCA level (Figure 4B). In order to investigate the impact of *A. muciniphila* on FGF15/19, we therefore measured ileum *FGF15* expression in mice treated with *A. muciniphila*. We found significantly reduced *FGF15* mRNA levels in HS diet‐fed mice compared with mice fed with normal chow (**Figure** **6**A). Treatment with live *A. muciniphila* restored the diminished *FGF15* level caused by the HS diet (Figure 6A). In addition, liver glycogen content was significantly reduced with an HS diet but increased after treatment with live *A. muciniphila* (Figure 6B). The increased gluconeogenesis with an HS diet, as determined by RT‐PCR analysis of the expression levels of relevant genes, was attenuated by supplementation with *A. muciniphila* (Figure 6C).

FGF19 expression can be regulated by BA‐mediated activation of FXR,^[^ ^20^, ^39^ ^]^ and CDCA has been found to induce FGF19 expression significantly.^[^ ^40^ ^]^ To further study if *β*CDCA could inhibit FGF15/19 expression, the human epithelial colon cell line LS174T was cultured with various concentrations of *β*CDCA in the presence of CDCA. We found that treatment of LS174T cells with CDCA together with *β*CDCA caused a dose‐dependent decrease of FGF19 mRNA expression levels (Figure 6D), suggesting the inhibitory effect of *β*CDCA on intestinal *FGF15/19* expression. The above findings indicate that *A. muciniphila* might enhance the expression of FGF15/19 and cause the subsequent stimulation of glycogen synthesis and inhibition of gluconeogenesis, at least partially, by limiting the availability of *β*CDCA.

## 3 Discussion and Conclusion

**Figure 7:**
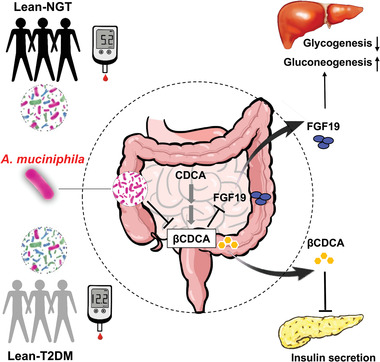
Proposed mechanism of action for *A. muciniphila* on glucose metabolism by improvement of insulin secretion and FGF19 expression. *A. muciniphila* abundance in the gut was depleted in the lean diabetic gut microbiome, which was associated with the increase of *β*CDCA and reduction of insulin secretion and FGF19 expression. Furthermore, *A. muciniphila* treatment modulates *β*CDCA levels then stimulates insulin secretion and FGF19 expression, thus leading to improved glucose metabolism. *A. muciniphila, Akkermansia muciniphila*; *β*CDCA, 3*β*‐chenodeoxycholic acid; CDCA, chenodeoxycholic acid; FGF19, fibroblast growth factor 19.

In the present study, we exploited shotgun metagenomic sequencing data from participants with different glucose tolerance states and degrees of abdominal obesity. We showed that *A. muciniphila* abundance was depleted in the gut microbiota of lean participants with T2D and this was associated with a reduction in insulin secretion and FGF19 expression. Furthermore, supplementation with *A. muciniphila* protected mice against diet‐induced glucose intolerance. Our investigation into the mechanisms behind the beneficial impact of *A. muciniphila* on host metabolism revealed that *A. muciniphila* treatment modulated *β*CDCA levels, then stimulated insulin secretion and FGF19 expression, thus leading to reduced blood glucose levels and improved glucose homeostasis (**Figure** **7**).

This study reports a specific microbiota signature in lean individuals with newly diagnosed T2D. More than 80% of people with T2D are overweight or obese,^[^ ^2^, ^3^ ^]^ however, a recent study found that the patients with T2D who are obese and lean have different characteristics and risks of diabetic complications.^[^ ^6^ ^]^ Even though prior studies have demonstrated that altered gut microbiota is linked to T2D,^[^ ^11^, ^12^, ^13^ ^]^ most of these studies did not differentiate between individuals with T2D who were lean or obese. Whether lean individuals have a unique gut microbiome composition remained unclear. In our investigation, a clear separation for overall gut microbiota between the T2D‐NO and T2D‐O groups, as well as between the T2D‐NO and NGT‐O groups was observed (Figure 2). Interestingly, in our Chinese cohort the NGT‐O and T2D‐O (newly diagnosed T2D) groups shared highly similar gut microbial community profiles, in agreement with a recent study on a German population. In that study, significant differences were not observed between obese participants with and without T2D for microbial taxonomic or functional profiles after adjusting for diabetic medications.^[^ ^41^ ^]^ These results imply a possible transitional stage from NGT‐O to T2D‐O, given the close relationship between obesity and T2D, as well as the crucial role of gut microbiota in the pathogenesis of both diseases.

Among the differentially abundant species between groups, we found the abundance of *A. muciniphila* was decreased in T2D‐NO participants. *A. muciniphila* was reported to have a higher abundance in an NGT group than a prediabetic/diabetic group^[^ ^42^, ^43^ ^]^ and supplementation with *A. muciniphila* tends to improve glucose tolerance in high‐fat diet mice.^[^ ^33^, ^44^ ^]^ A clinical trial discovered that supplementation with pasteurized *A. muciniphila* in overweight or obese participants reduces plasma total cholesterol.^[^ ^45^ ^]^ Our study demonstrated that supplementation of lean, glucose‐intolerant mice with *A. muciniphila* improved glucose tolerance by improving insulin secretion, suggesting that therapeutic interventions targeting this single species in the gut microbiota may be a promising strategy for the treatment and prevention of diabetes in lean individuals.

Our study shed light on the positive relationship between *A. muciniphila* and insulin secretion and FGF19. FGF19 and its mouse ortholog FGF15 show multiple benefits on glucose homeostasis through insulin‐independent pathways.^[^ ^38^, ^46^, ^47^ ^]^ We did not observe a significant correlation between FGF19 and insulin secretion or insulin sensitivity, consistent with our previous research.^[^ ^48^ ^]^ Previous studies demonstrated that both insulin and FGF19 participate in hepatic glucose regulation.^[^ ^46^, ^49^ ^]^ We found that the liver glycogen content in the HS‐water group decreased significantly compared with control mice, and treatment of HS mice with *A. muciniphila* increased liver glycogen levels. In addition, expression of genes involved in gluconeogenesis increased in the HS‐water group and partially returned to control levels upon *A. muciniphila* treatment. We propose that *A. muciniphila* promotes insulin secretion and expression of FGF15/19, which then induces glycogen synthesis and suppresses gluconeogenesis, thus improving glucose tolerance.

Interestingly, we demonstrated that *β*CDCA levels increased in the T2D‐NO participants and was inversely associated with gut microbiota *A. muciniphila* and *baiB* gene abundances, insulin secretion and FGF19 levels. The product of *baiB* gene is responsible for ligating primary BAs to coenzyme A^[^ ^50^, ^51^ ^]^ and its abundance was found to be positively correlated with intestinal *A. muciniphila* abundance. Previous studies indicated that gut microbes influence biotransformation of BAs including deconjugation, oxidation‐reduction, dehydroxylation, and hydroxylation through changing the relative abundance of microbial genes involved in BA metabolism.^[^ ^52^, ^53^ ^]^ Through analyzing the KEGG secondary BA biosynthesis pathway, we observed two different directions between T2D‐NO and NGT‐NO group. Compared with the T2D‐NO group, individuals in the NGT‐NO group had higher abundances of *A. muciniphila* and *baiB*, lower levels of *β*CDCA, and increased insulin secretion and FGF19 levels, which is in keeping with the inhibitory effects of *β*CDCA on insulin secretion and FGF19 expression demonstrated by our in vitro experiments. The results indicated that *A. muciniphila* may modulate insulin secretion and FGF19 expression through reducing the pool of the bile acid *β*CDCA.

Limitations of this study are that larger sample sizes in the T2D‐NO group are needed to further characterize the gut microbiota of lean participants with T2D and to verify our findings. In addition, detailed food frequency questionnaires should be included to account for the dietary effect. In animal experiment, high‐sucrose diet might also induce hypertension and dyslipidemia,^[^ ^36^, ^54^ ^]^ which might be a limitation in modeling the metabolic dysfunction in human. Further studies are warranted to address the possible molecular mechanism underlying the influence of *A. muciniphila* on *β*CDCA and insulin secretion.

In summary, our work demonstrated the close relationships between changes of specific gut microbes and host glucose metabolism in lean participants with T2D. We further showed that *A. muciniphila* modulates insulin secretion and FGF19 expression through altering *β*CDCA levels. These findings suggest an additional mechanism for improving glucose tolerance with a single gut microbiota member, *A. muciniphila*, paving the way for future human studies investigating *A. muciniphila* as a therapeutic tool in the management of lean individuals with T2D.

## 4 Experimental Section

### Study Participants

Based on the Shanghai Nicheng Cohort Study,^[^ ^29^ ^]^ a subset of 182 participants representing four groups of gender‐matched adult individuals was selected from May 2014 to June 2014. OGTT was performed for all participants. The diagnosis of various glucose tolerance status was based on the 2003 American Diabetic Association diagnostic criteria.^[^ ^55^ ^]^ FPG < 6.1 mmol L^−1^ and 2hPG < 7.8 mmol L^−1^ were classified as NGT. T2D was defined as FPG ≥ 7.0 mmol L^−1^ and/or 2hPG ≥ 11.1 mmol L^−1^. Visceral obesity was defined as VFA ≥80 cm^2^.^[^ ^56^ ^]^ Participants with the following conditions were excluded: type 1 diabetes, acute infectious disease, biliary obstructive diseases, alcoholic abuse, acute or chronic cholecystitis, acute or chronic viral hepatitis, cirrhosis, diarrhea, known hyperthyroidism or hypothyroidism, chronic renal insufficiency, heart failure, presence of cancer, pregnancy, stroke in acute phase, gastrointestinal disease and gastrointestinal surgery within 5 years before recruitment, receipt of any antibiotic treatment within 3 months before sample collection, regular use of a probiotic or prebiotic supplement within 6 weeks prior to enrollment, current treatment with BAs and BA sequestrants, and use of drugs that affect insulin secretion and sensitivity. All participants underwent comprehensive physical examinations with routine biochemical blood analyses and electrocardiograms. Information on demographics, smoking habits, alcohol consumption, educational level, family history of diseases, history of surgery, and current and past medical therapy was obtained through a uniform questionnaire. The study complied with the Declaration of Helsinki and was approved by the local ethics committee. All the participants gave informed consent.

### Clinical and Biochemical Measurements

Plasma glucose levels were quantified by the hexokinase method. Measurement of SFA and VFA was determined by magnetic resonance imaging. Serum insulin levels were assayed by radioimmunoassay (Linco Research, St. Charles, MO). Basal insulin secretion and insulin sensitivity were assessed by HOMA‐%B and HOMA‐IR using equations HOMA‐%B = (fasting insulin levels (FINS) (mU L^−1^) × 6 × 3.33]/[FPG (mmol L^−1^) − 3.5) and HOMA‐IR = FINS (mU L^−1^) × FPG (mmol L^−1^)/22.5.^[^ ^57^ ^]^ ISICederholm was also used for assessing insulin sensitivity, ISICederholm = [75 000 + (FPG − 2hPG) × 1.15 × 180 × 0.19 × body weight]/[120 × log(mean insulin) × mean glucose].^[^ ^58^ ^]^ 1st PH was calculated as 1283 + 1.829 × (serum insulin levels at 30 min at OGTT (Ins30) (pmol L^−1^)) – 138.7 ×(plasma glucose levels at 30 min at OGTT (Gluc30) (mmol L^−1^)) + 3.772 × FINS, and 2nd PH was calculated as 286 + 0.416 × Ins30 (pmol L^−1^) – 25.94 × Gluc30 (mmol L^−1^) + 0.926 × FINS.^[^ ^59^ ^]^ Serum FGF19 levels were determined using enzyme‐linked immunosorbent assay (ELISA) kits (Antibody and Immunoassay Services, University of Hong Kong). Glutamic acid decarboxylase antibodies (EUROIMMUN) and insulin autoantibody were measured by ELISA (Biomerica).

### Fecal Sample Collection, DNA Extraction, and Metagenomic Sequencing

Fecal samples were collected using tubes with DNA stabilizer (STRATEC Molecular, Germany) and stored at −80 °C. Stool DNA was extracted using PSP Spin Stool DNA kits (STRATEC Molecular, Germany) following the manufacturer's instructions. Extracted DNA was subjected to shotgun metagenomic sequencing on an Illumina HiSeq 4000 platform with 150 bp paired‐end reads at BGI (Hong Kong S.A.R., China).

### Metagenomics Quality Control and Taxonomy Profiling

Average sequencing throughput for each sample (total *n* = 182) was around 31.6 million reads (range, 21 million to 40 million). In‐house Perl scripts were used to remove adaptors, low‐quality reads, bases, and PCR duplicates as previously described.^[^ ^60^ ^]^ Human‐derived reads were removed using BWA‐mem^[^ ^61^ ^]^ (version 0.7.4) and a human reference genome (ucsc.hg19). After quality control and filtering, 31.2 million reads per sample on average remained and were used in downstream analyses.

MetaPhlAn2^[^ ^62^ ^]^ was employed for community taxonomy profiling at different taxonomic levels with the default setting, generating taxonomic relative abundances (total sum scaling normalization). R package vegan^[^ ^63^ ^]^ was used to calculate the Shannon and Simpson alpha diversity for each sample based on the relative abundance of genera. To deduce community dissimilarity between samples (beta diversity), the UniFrac distance (weighted and unweighted) calculated by phyloseq was used.^[^ ^64^ ^]^ Co‐abundance network analysis was conducted using SparCC^[^ ^65^ ^]^ for species present in at least 20% of samples (100 inference iterations for correlation estimation and 100 permutations for pseudo *p* value calculation). Only correlations larger than 0.3 or smaller than −0.3 were used for network construction and visualization.

### De Novo Assembly, Gene Prediction, Functional Annotation, and Analysis

Paired‐end reads were assembled using IDBA‐UD^[^ ^66^ ^]^ with k‐mer size 20 to 150 bp. Contigs less than 300 bps were discarded from further analysis. MetaGeneMark^[^ ^67^ ^]^ was adopted to predict the coding sequence regions in the assembled metagenome contigs using default parameters. The functional category from clusters of orthologous groups^[^ ^68^ ^]^ for each protein was assigned using NCBI RPS‐BLAST against the conserved domains database (v.3.10) at 1e‐5 cutoff, with KEGG Orthology (KO) annotation through the combinatorial use of DIAMOND^[^ ^69^ ^]^ and KOBAS 2.0 annotate programs.^[^ ^70^ ^]^ In addition, PRIAM (with default parameters)^[^ ^71^ ^]^ was used to annotate genes to enzyme commission (ECs) numbers that were further used for profiling MetaCyc pathways.^[^ ^72^ ^]^ Bray–Curtis dissimilarity was used to evaluate functional diversity between samples. KEGG pathway and functional module abundances were estimated by summing the abundances of all genes in the corresponding pathways and modules, respectively (KEGG accessed August 2017). Differentially altered KEGG pathways and modules were identified using Wilcoxon rank‐sum test.

### Targeted Metabolomics Quantification of Serum BAs and BCAA

Metabo‐Profile Inc. (Shanghai, China) performed BA profiling and quantitation using published methods with modifications.^[^ ^73^, ^74^, ^75^ ^]^ Serum BAs were assayed using an ultraperformance liquid chromatography coupled to a tandem mass spectrometry (UPLC‐MS/MS) system (ACQUITY UPLC‐Xevo TQ‐S, Waters Corp., Milford, MA, USA). Solvent A was formic acid/water (pH = 3.25), and solvent B was acetonitrile/methanol (80:20, v/v). The gradient was set as follows: 5% B at 0–1 min, 5–30% B at 1–3 min, 30–100% B at 3–15 min, 100–5% B at 15–16 min, and 5% B at 16–17 min. The flow rate was set to 0.4 mL min^−1^. Chromatographic separation was performed on a 2.1 × 5 mm ACQUITY UPLC Cortecs C18 1.6 × 10^−6^ m VanGuard pre‐column and a 100 mm × 2.1 mm ACQUITY UPLC Cortecs C81.6 × 10^−6^ m analytical column. Sample preparations for BA assays were as described previously.^[^ ^73^, ^74^, ^75^ ^]^ A series of standard calibration solutions were diluted with desalted serum (depleted of BAs using activated charcoal) for the calibration curve.^[^ ^76^ ^]^ The concentration of BA in an unknown sample was determined by comparing the unknown to calibration curve.

Metabo‐Profile Inc. (Shanghai, China) performed BCAA quantitation using published methods with modifications.^[^ ^77^, ^78^ ^]^ Serum BCAA were measured using a quantitative gas chromatography coupled to time‐of‐flight mass spectrometry (GC‐TOFMS) system (Pegasus HT, Leco Corp., St. Joseph, MO, USA). The system utilized an Rxi‐5Sil MS column coated with 5% diphenyl crosslinked with 95% dimethylpolysiloxane (30 m × 250 µm inner diameter, 0.25 µm film thickness, RESTEK, PA, USA), with helium as the carrier gas at a flow rate of 1.0 mL min^−1^. Each 1 µL aliquot of the analyte was injected in splitless mode. The initial temperature was kept at 80 °C for 2 min, and then raised to 300 °C at a rate of 12 °C min^−1^, and a final 8 min maintenance at 300 °C. The injection, transfer line, and ion source temperatures were 270, 270, and 220 °C, respectively. The energy was 70 eV in electron impact mode. The mass spectrometry data were acquired in full‐scan mode with the *m*/*z* range of 50–550 at a rate of 25 spectra per second after a solvent delay of 264 s. A calibration curve was also used for determining BCAA concentration.

### Animal Studies

Animal experimental procedures were approved by the Committee on the Use of Live Animals for Teaching and Research of the Shanghai Dunwill Medical Technology Animal Research Center (SYXK (Shanghai) 2016‐0015). All relevant ethical regulations were complied. Eight‐week‐old C57BL/6J‐mice (Nanjing Biomedical Research Institute of Nanjing University, China) were kept in a specific pathogen‐free barrier facility in individually ventilated cages with five mice per cage. Water was given ad libitum. Animals were fed either a normal chow diet or HS diet (containing 42% sucrose [g%]^[^ ^35^ ^]^). Animals on the HS diet were randomized into three groups with their sterile drinking water fortified with: *A. muciniphila*, heat‐killed *A. muciniphila*, or nothing for 15 weeks. Drinking water was changed daily. Body weight was measured every 7 days.

*A. muciniphila* (catalog No. 22959, Type strain, DSMZ, German) was cultured anaerobically in brain‐heart‐infusion broth (BD Bioscience, San Jose, CA) supplemented with 0.5% porcine mucin (Sigma‐Aldrich, St. Louis, MO) and 0.05% cysteine (Sigma‐Aldrich) as previously described.^[^ ^79^ ^]^ Culture density was calculated by measuring absorbance at wavelength 600 nm. Culture purity was monitored by Gram staining and CFUs were counted by plating serial dilutions on agar plates. *A. muciniphila* was collected by centrifuging at 3000 rpm for 30 min at 4 °C, washing with sterile PBS twice and resuspending to 5 × 10^10^ CFUs mL^−1^ in 2 mL anaerobic sterile PBS containing 20% glycerol and storing at −80 °C. For the hk‐AKK group, *A. muciniphila* were heat killed at 121 °C under 225 kPa pressure for 15 min.

### Glucose Tolerance Test (GTT), Insulin Tolerance Test (ITT), and Glucose‐Stimulated Insulin Secretion (GSIS)

For GTT, mice were fasted overnight, and glucose (1 g kg^−1^ body weight) was injected intraperitoneally. Blood glucose levels were determined from tail vein samples at fasting state and 10, 20, 30, 45, 60, 90, and 120 min after initial glucose injection.

For ITT, mice were fasted for 6 h, and insulin (1 U kg^−1^ body weight) was injected intraperitoneally. Blood glucose levels were determined from tail vein samples at fasting state and 15, 30, 60, 90, and 120 min after initial insulin injection.

For GSIS, mice were fasted overnight, and glucose (3 g kg^−1^ body weight) was injected intraperitoneally. The blood from tail veins was sampled at fasting state and 15 min after initial glucose injection. Serum insulin levels were measured using insulin ELISA kits (Antibody and Immunoassay Services, The University of Hong Kong).

### Cell Cultures

The mouse pancreatic beta cell line MIN6 (gift from Dr. Feng Liu, Department of Pharmacology, University of Texas Health Science Center at San Antonio) was used for studying the influence of *β*CDCA on insulin secretion. Cells were grown in Dulbecco's Modified Eagle Medium containing 15% fetal bovine serum, 1% penicillin–streptomycin, 2.5 g L^−1^ NaHCO_3_, 0.11 g L^−1^ sodium pyruvate, 2 g L^−1^ HEPES, and 50 × 10^−6^ m beta‐mercaptoethanol. Cultures were maintained at 37 °C in a humidified 5% CO_2_ atmosphere. MIN6 cells were stimulated for 1 h with 2.8, 8.3, and 15 mmol L^−1^ glucose in the presence or absence of 0.5 µmol L^−1^ *β*CDCA (Toronto research chemicals).

The human intestinal cell line LS174T (Chinese Cell Bank of the Chinese Academy of Sciences) was used for studying the influence of *β*CDCA on FGF19 expression. CDCA significantly induces FGF19 expression in LS174T cells.^[^ ^40^ ^]^ Cells were grown in minimal essential medium containing 10% FBS and penicillin–streptomycin. Cultures were maintained at 37 °C in a humidified 5% CO_2_ atmosphere. LS174T cells were stimulated for 24 h with 50, 100, and 200 µmol L^−1^ *β*CDCA in the presence of 150 µmol L^−1^ CDCA (Sigma).

### Quantitative RT‐PCR

Total RNA was prepared using TRIzol reagent (Ambion). RT‐PCR was performed with a Roche Lightcycler 96 system, using FastStart Universal SYBR Green Master (ROX) (Roche). Relative mRNA levels were calculated by the comparative threshold cycle method. Primer sequences are in Table S5 in the Supporting Information.

### Islet Isolation and Insulin Secretion Assays

Mouse pancreatic islets were isolated as published with minor modifications.^[^ ^80^, ^81^ ^]^ After ductal distension of the pancreas and tissue digestion with collagenase P (Roche Applied Science), pancreas samples were digested at 37 °C for 20 min and filtered through cell strainers, resulting in two fractions: flow‐through containing exocrine cells (nonislet fraction) and captured fraction (islets). The captured fraction was density‐gradient centrifuged with Histopaque 1077 and Histopaque 1119 (Sigma). Islets were picked manually under a microscope and maintained in Ham's F‐12K medium supplemented with 10% (v/v) FBS at 37 °C overnight. Isolated islets were washed twice with Krebs Ringer bicarbonate buffer containing 0.1% fatty acid‐free bovine serum albumin supplemented with 2.8 × 10^−3^ m glucose for 1 h, followed by stimulation with different concentrations of glucose. Insulin secreted into the supernatant was measured using an insulin ELISA kit (Antibody and Immunoassay Services, The University of Hong Kong), and normalized for the number of islets.

### Statistical Analysis

For the clinical data, normally distributed data were expressed as mean ± SD. Data that were not normally distributed, as determined by the Shapiro–Wilk test, were logarithmically transformed before analysis and expressed as median with interquartile range. For the data of animals or cell lines, all values were expressed as means ± SEM. The relevant sample sizes are described in the corresponding figures and tables. Single comparison between two groups was analyzed by Student's *t*‐test. Chi‐squared test and one‐way analysis of variance were used for comparisons of categorical and continuous data among groups, respectively, and multiple testing was corrected using Bonferroni post hoc test or Fisher's LSD post hoc test. For the metagenomics data, comparisons of microbiota alpha diversity, species‐level relative abundances and functional categories were conducted using Wilcoxon rank‐sum test. Differential abundance analyses comparing T2D‐NO group with each of the other three groups were performed using one‐sided Wilcoxon rank‐sum test to assess for significant elevation or depletion. Permutational multivariate analysis of variance with “adonis” function in R package vegan was used for microbiota beta diversity comparison. Correlation analysis was conducted using Spearman's rank correlations or partial spearman correlations adjusting for age. SPSS version 16.0 and R software were used for statistical analyses. Two‐tailed *p* values <0.05 were considered significant unless otherwise stated. Multiple hypothesis testing correction was performed with the false‐discovery rate method using R package stats.

## Conflict of Interest

The authors declare no conflict of interest.

## Author Contributions

J.Z., Y.N., L.Q., and Q.F contributed equally to this work. W.J., G.P., and H.L. conceived and designed the project. J.Z., Y.N., and L.Q. managed the study. J.Z. and L.Q. collected samples and extracted DNA from feces. J.Z., L.Q., and X.H. collected clinical phenotypes. Y.N., T.Z., and G.P. performed bioinformatics analyses. J.Z., M.Z., and Q.F conducted animal experiments. Q.G., Y.Z., and J.N. conducted experiments on measuring circulating BCAA. J.Z. and Y.N. wrote the manuscript. W.J., G.P., Y.B., and H.L. reviewed and edited the manuscript. All authors made substantial contributions and approved the final version of the manuscript.

## Supporting information

Supporting Information

## Data Availability

The raw metagenomic sequencing data for all samples have been deposited in NCBI Sequencing Read Archive under BioProject ID PRJNA686835. Further information and requests for resources and reagents should be directed to Weiping Jia (wpjia@sjtu.edu.cn).
