# Supplementary material for: Decreased Abundance of Akkermansia muciniphila Leads to the Impairment of Insulin Secretion and Glucose Homeostasis in Lean Type 2 Diabetes
Source: Adv Sci (Weinh). 2021 Jun 4;8(16):2100536. doi: 10.1002/advs.202100536 (PMC8373164; doi:10.1002/advs.202100536)
Supplement: Supplementary file 1 — Supporting Information [file ADVS-8-2100536-s001.pdf]

© 2021 Wiley-VCH GmbH

ADVANCED SCIENCE Open Access

Supporting Information

for *Adv. Sci.,* DOI: 10.1002/advs.202100536

Decreased Abundance of *Akkermansia muciniphila* Leads to the Impairment of Insulin Secretion and Glucose Homeostasis in Lean type 2 Diabetes

Jing Zhang, Yueqiong Ni, Lingling Qian, Qichen Fang, Tingting Zheng, Mingliang Zhang, Qiongmei Gao, Ying Zhang, Jiacheng Ni, Xuhong Hou, Yuqian Bao, Petia Kovatcheva-Datchary, Aimin Xu, Huating Li, Gianni Panagiotou*, Weiping Jia**

Supplementary Information

Decreased abundance of *Akkermansia muciniphila* leads to the impairment of insulin secretion and glucose homeostasis in lean type 2 diabetes

Jing Zhang, Yueqiong Ni, Lingling Qian, Qichen Fang, Tingting Zheng, Mingliang Zhang, Qiongmei Gao, Ying Zhang, Jiacheng Ni, Xuhong Hou, Yuqian Bao, Petia Kovatcheva-Datchary, Aimin Xu, Huating Li, Gianni Panagiotou*, Weiping Jia**

(J.Z., Y.N., L.Q., and Q.F contributed equally to this work)

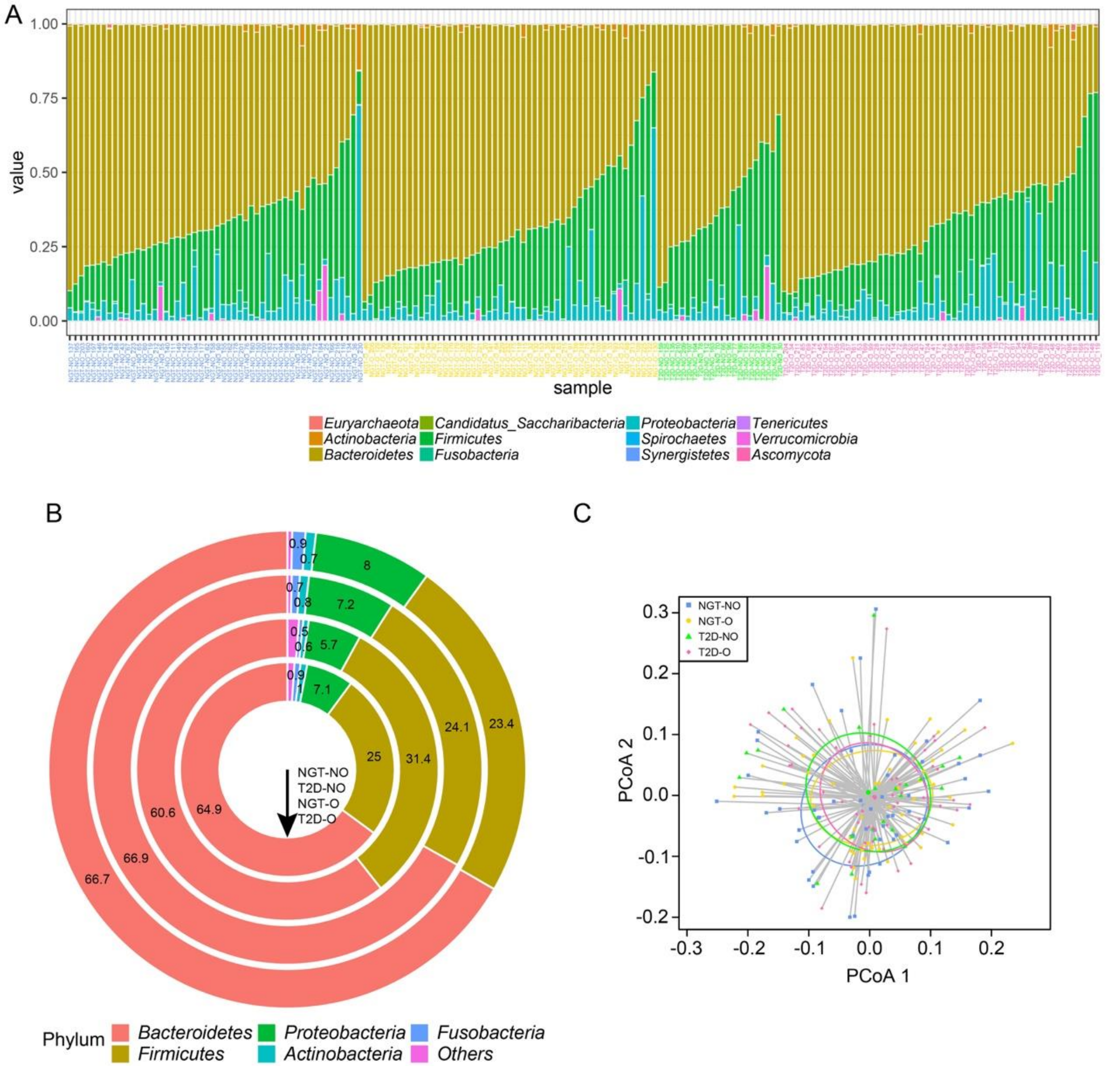

Figure S1. Taxonomic profiles of the gut microbiota for the four groups. (A) The taxonomic composition of the gut microbiota at the phylum level for all the samples. Samples were ordered and colored based on the affiliated groups: blue, NGT-NO; yellow, NGT-O; green, T2D-NO; pink, T2D-O. (B) The average relative abundances of gut microbiota phyla for the four groups shown as donut plot. The mean abundance of each phylum (only the top five most abundant) within each group was marked as percentage. Inner to outer rings: NGT-NO, T2D-NO, NGT-O and T2D-O. (C) Principal Coordinate Analysis (PCoA) plot based on weighted UniFrac distance for 4 different groups. NGT-NO, normal glucose tolerance-lean; NGT-O, normal glucose tolerance-abdominally obese; T2D-NO, type 2 diabetes-lean; T2D-O, type 2 diabetes-abdominally obese.

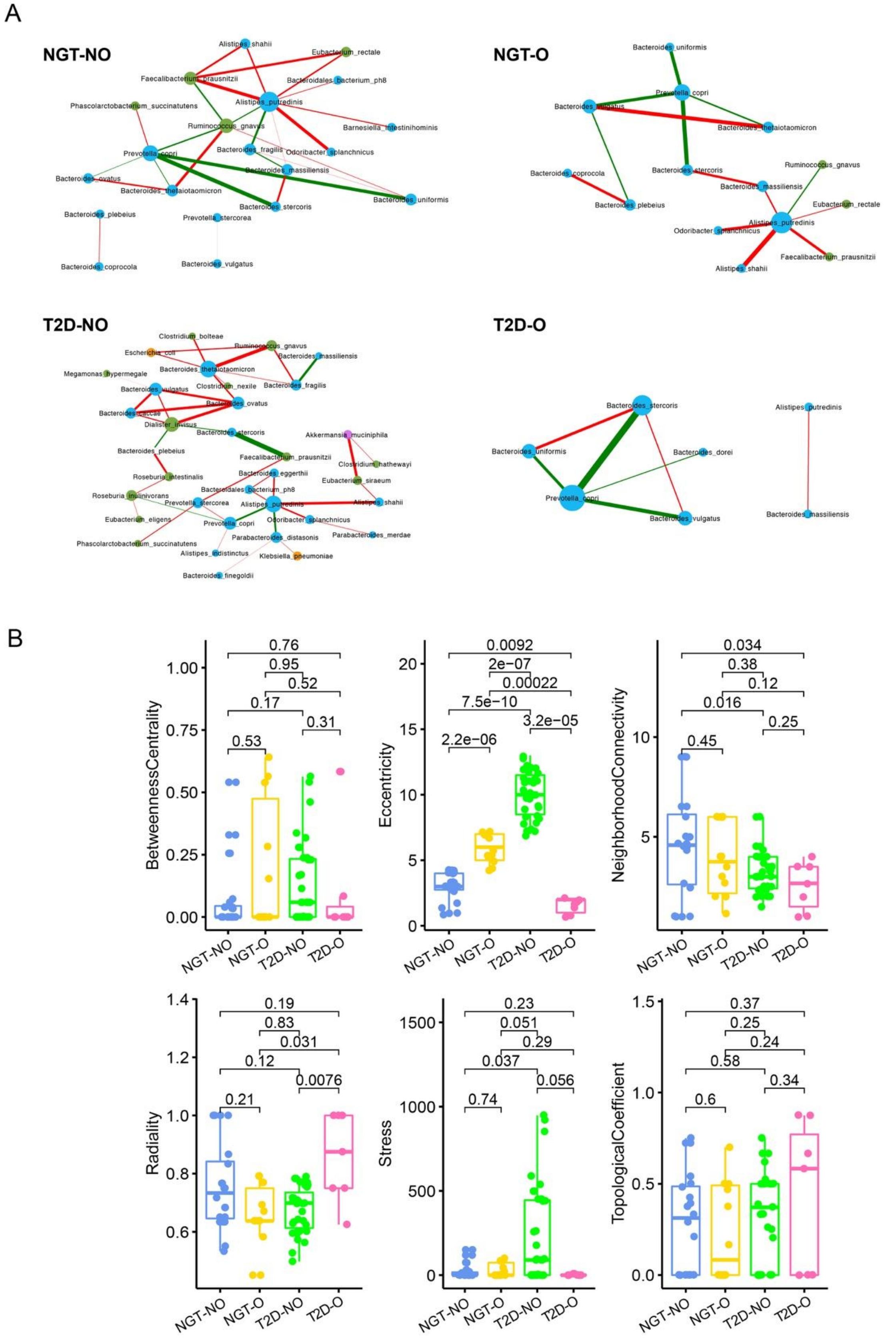

Figure S2. Bacteria-bacteria co-abundance networks in different groups. (A) Microbial co-abundance was calculated with Spearman’s rank-based correlations for prevalent species (present in more than 50% of the samples) within each group. The edges represent significantly (P<0.05) positive

(red) or negative (green) correlations and the edge width is proportional to the absolute value of correlations. Nodes are colored based on their affiliated phyla and the node size is proportional to the average relative abundance of species. (B) Comparison of co-abundance network topological properties for the four groups. Statistical p values determined from Wilcoxon rank-sum tests are marked in pairwise manner. Boxplots show median (centerlines), lower/upper quartiles (box limits), whiskers (the last data points 1.5 times IQR from the lower or upper quartiles), and notches (95% confidence interval for the medians). NGT-NO, normal glucose tolerance-lean; NGT-O, normal glucose tolerance-abdominally obese; T2D-NO, type 2 diabetes-lean; T2D-O, type 2 diabetes-abdominally obese.

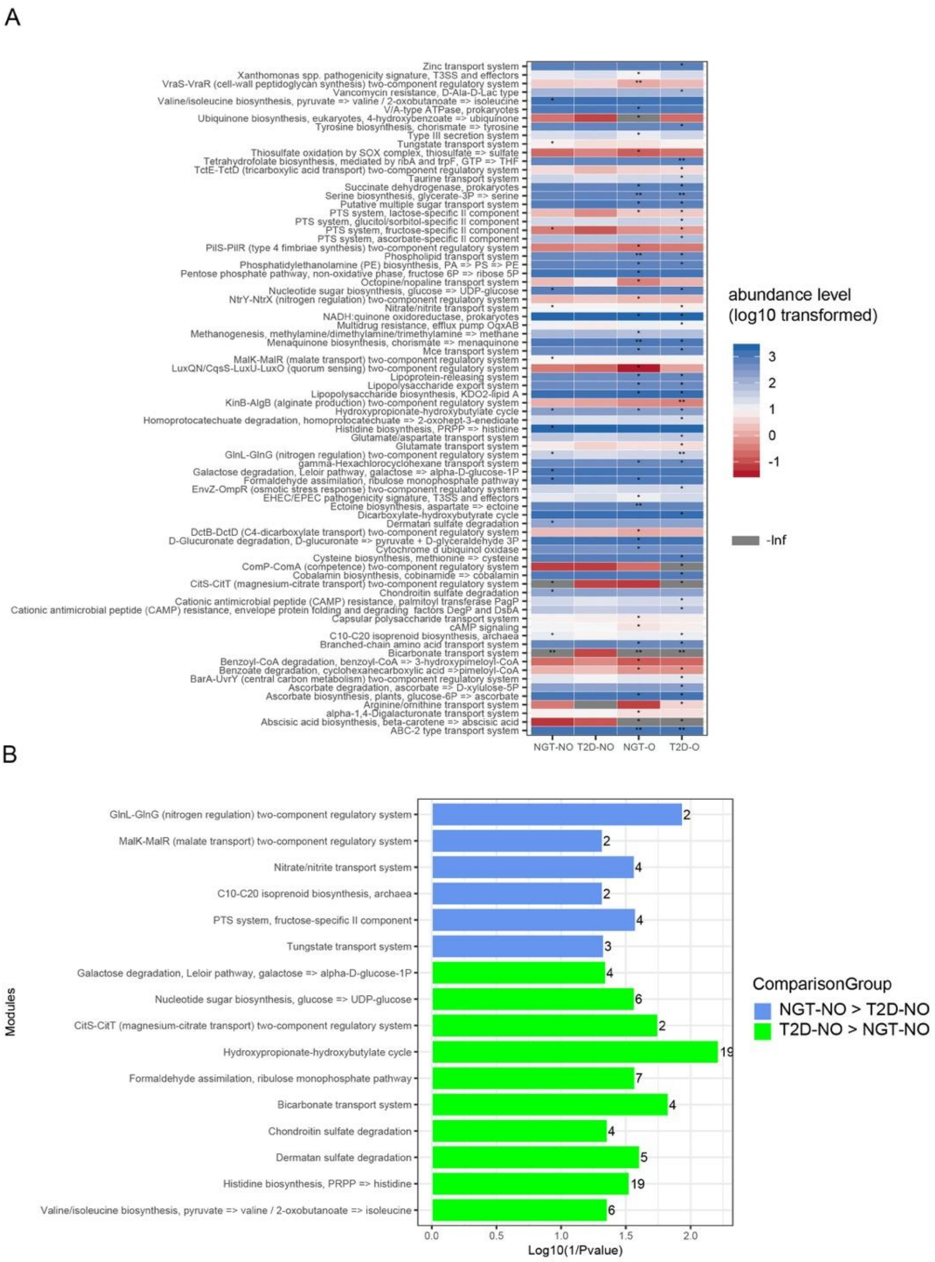

Figure S3. Gut microbiota KEGG module enrichment analysis. (A) Differentially abundance gut microbial KEGG modules comparing the T2D-NO group against each of the other three groups. *, P < 0.05, Wilcoxon rank-sum test when comparing against T2D-NO. For visualization purpose, the relative abundances of functional modules were log10-transformed. (B) KEGG modules significantly enriched in either NGT-NO (blue) or T2D-NO (green) groups. The number of KEGG Orthologs within each KEGG module was marked along the corresponding bar. NGT-NO, normal glucose tolerance-lean; T2D-NO, type 2 diabetes-lean

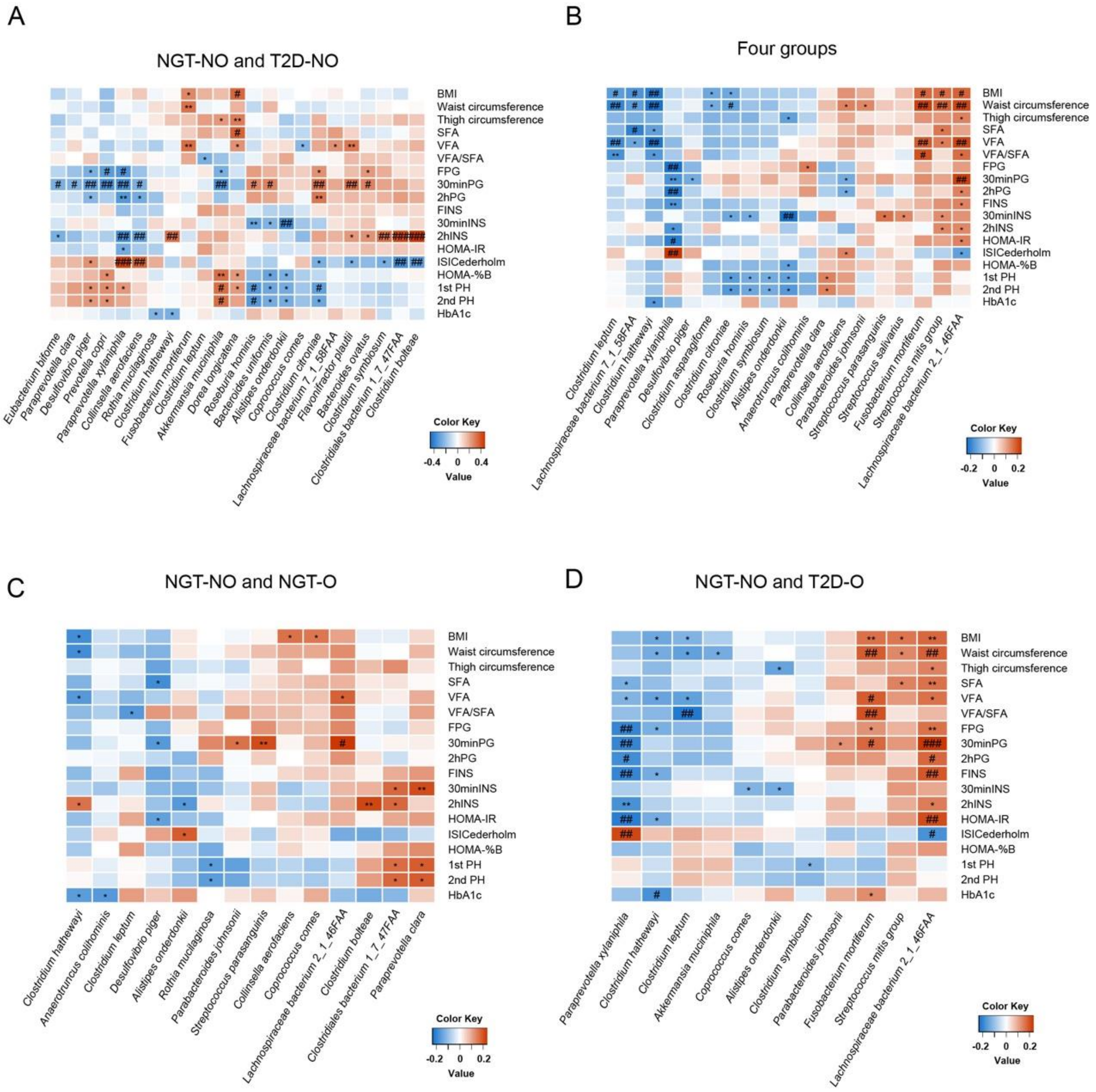

Figure S4. Correlations between specific gut bacteria and clinical indices among different groups. Spearman's rank-based correlations were calculated between prevalent species (>30% in at least one group) and clinical metadata for samples from (A) T2D-NO and NGT-NO groups; (B) all four groups; (C) NGT-O and NGT-NO groups; (D) T2D-O and NGT-NO groups. Only species with at least one significant correlation ($P < 0.05$) were included. Red, positive correlations; blue, negative correlations. #, FDR-corrected P< 0.1; ##, FDR-corrected P <0.05; ###, FDR-corrected P< 0.01; *, P< 0.05; **, P< 0.01; ***, P< 0.001. BMI, body mass index; FINS, fasting serum insulin; FPG, fasting plasma glucose; HOMA-%B, homeostasis model assessment of insulin secretion; HOMA-IR, homeostasis model assessment of insulin resistance; 30min PG, 30min plasma glucose during OGTT; 30min INS, 30min serum insulin during OGTT; 2hPG, 2-h plasma glucose during OGTT; 2hINS, 2-h serum insulin during OGTT; NGT-NO, normal glucose tolerance-lean; NGT-O, normal glucose tolerance-abdominally obese; SFA, subcutaneous abdominal fat area; T2D-NO, type 2 diabetes-lean; T2D-O, type 2 diabetes-abdominally obese; VFA, visceral abdominal fat area. 1st PH, first-phase insulin release; 2nd PH, second-phase insulin release.

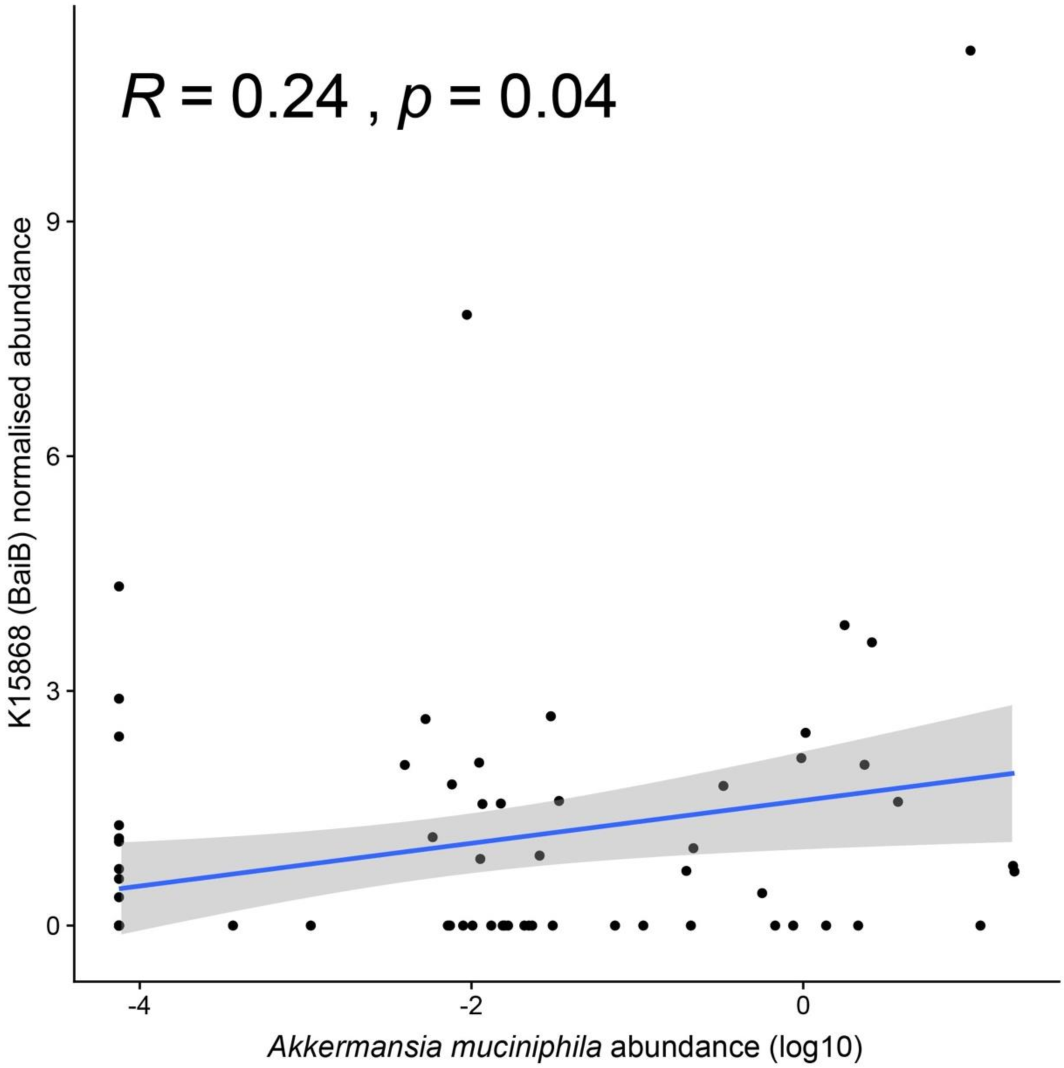

Figure S5. Scatter plot showing the correlation between *Akkermansia muciniphila* and gut microbial gene K15868. All samples from NGT-NO and T2D-NO groups were used for Spearman's rank correlation estimation. For visualization purpose, the relative abundances of Akkermansia muciniphila were log10-transformed. The abundance of bacterial gene K15868 was quantified in an RPKM-like manner (normalized by both the library size and the gene length). NGT-NO, normal glucose tolerance-lean; T2D-NO, type 2 diabetes-lean.

Table S1 Statistical comparison of microbial community alpha and beta diversity

Comparisons	Alpha diversity			Beta diversity (Weighted UniFrac)	
	Simpson	Shannon	Evenness	R^2	P
**NGT-NO vs T2D-NO**	0.527	0.474	0.46	0.023	0.108
**NGT-NO vs NGT-O**	0.723	0.587	0.619	0.006	0.733
**NGT-NO vs T2D-O**	0.107	0.096	0.066	0.009	0.424
**T2D-NO vs NGT-O**	0.411	0.335	0.274	0.039	0.030
**T2D-NO vs T2D-O**	0.041	0.049	0.02	0.045	0.012
**NGT-O vs T2D-O**	0.324	0.419	0.292	0.003	0.918

Statistical comparisons of alpha diversity and beta diversity (weighted UniFrac distances) were performed using Wilcoxon rank-sum test and permutational multivariate analysis of variance (PERMANOVA), respectively. NGT-NO, normal glucose tolerance-lean; NGT-O, normal glucose tolerance-abdominally obese; T2D-NO, type 2 diabetes-lean; T2D-O, type 2 diabetes-abdominally obese.

Table S2 Anthropometric parameters and biochemical indexes among the subgroup for investigating serum BAs (n=80)

Variables	NGT-NO (n=20)	NGT-O (n=20)	T2D-NO (n=20)	T2D-O (n=20)	*P* (subgroup vs. total cohort)				*P* (Four Groups)
					NGT-NO	NGT-O	T2D-NO	T2D-O	
**Male/female**	10/10	10/10	10/10	10/10					-
**Age (year)**	55.2 ± 6.6	56.7 ± 5.6	59.4 ± 5.0	58.0 ± 7.5	0.62	0.63	0.77	0.95	0.182
**VFA (cm^2) §**	51.3 (41.2, 71.6)	138.0 (129.8, 171.3) *†	63.7 (41.1, 75.5)	184.0 (145.9, 213.9) *†	0.62	0.84	0.84	0.57	< 0.001
**SFA (cm^2) §**	82.8 (66.3, 91.3)	197.5 (142.4, 232.2) *†	77.6 (63.4, 91.5)	196.5 (170.0, 243.5) *†	0.19	0.89	0.62	0.09	< 0.001
**BMI (kg/m^2)**	20.8 ± 1.5	27.7 ± 2.1*†	21.5 ± 2.3	28.5 ± 2.7*†	0.29	0.95	0.36	0.59	< 0.001
**FPG (mmol/L) §**	5.6 (5.3, 5.7)	5.7 (5.4, 6.0) †	7.2 (6.7, 7.6)*	7.1 (6.8, 7.5)*‡	0.54	0.54	0.83	0.62	< 0.001
**2hPG (mmol/L) §**	6.4 (5.5, 7.0)	6.7 (5.7, 7.2) †	12.1 (9.3, 17.0) *	12.5 (11.6, 15.7) *‡	0.44	0.85	0.69	0.58	< 0.001
**HbA1c (%) §**	5.3 (5.1, 5.5)	5.4 (5.1, 5.7) †	5.9 (5.4, 6.9)*	6.1 (5.6, 6.3)*	0.49	0.61	0.86	0.65	0.001
**FINS (μU/mL) §**	3.6 (1.9, 5.3)	7.2 (6.0, 9.6)*†	4.3 (2.7, 6.4)	9.5 (8.0, 11.8)*†	0.55	0.08	0.45	0.05	< 0.001
**2hINS (μU/mL) §**	25.9 (17.9, 32.7)	31.6 (20.6, 56.4)	41.9 (22.6, 49.5)	81.3 (50.3, 93.1) *†‡	0.89	0.61	0.73	0.75	< 0.001
**HOMA-IR §**	0.8 (0.5, 1.3)	1.9 (1.5, 2.3)*	1.4 (0.9, 2.2) *	3.1 (2.6, 3.7)*†‡	0.59	0.06	0.48	0.03	< 0.001
**HOMA-%B §**	35.4 (17.1, 54.1)	71.1 (55.7, 82.5) *†	23.6 (16.1, 29.4)	52.3 (39.8, 56.9) *†	0.48	0.21	0.43	0.35	< 0.001
**1st PH**	569.0 ± 51.0	950.7 ± 130.2 †	-125.1 ± 85.1*	410.1 ± 153.0†‡	0.07	0.83	0.34	0.59	< 0.001

**2nd PH**	175.6 ± 10.7	271.4 ± 29.0*†	41.7 ± 16.9*	158.0 ± 30.6†‡	0.06	0.90	0.33	0.43	< 0.001

Data are means ± SD or median (interquartile range). Data are means ± SEM for 1st PH or 2nd PH. Statistical significance between subgroup and the corresponding whole group was determined with Student' *t* test. Statistical significance among four groups was determined with ANOVA with Bonferroni multiple-comparison analysis. **§** Log transformed before analysis. BMI, body mass index; FINS, fasting serum insulin; FPG, fasting plasma glucose; HOMA-%B, homeostasis model assessment of insulin secretion; HOMA-IR, homeostasis model assessment of insulin resistance; 2hPG, 2-h plasma glucose; 2hINS, 2-h serum insulin; NGT-NO, normal glucose tolerance-lean; NGT-O, normal glucose tolerance-abdominally obese; SFA, subcutaneous abdominal fat area; T2D-NO, type 2 diabetes-lean; T2D-O, type 2 diabetes-abdominally obese; VFA, visceral abdominal fat area. 1st PH, first-phase insulin release; 2nd PH, second-phase insulin release. * vs NGT-NO, $P < 0.05$; † vs T2D-NO, $P < 0.05$； ‡, vs NGT-O, $P < 0.05$.

Table S3 Bile acids and branched-chain amino acids levels among the subgroup (n=80)

Variables	NGT-NO (n=20)	NGT-O (n=20)	T2D-NO (n=20)	T2D-O (n=20)	*P*
**βUCA (nmol/L)§**	0.2 (0.1, 0.3)	0.7 (0.2, 1.6)*	0.5 (0.3, 1.3)*	0.4 (0.2, 1.0)	0.004
**UCA (nmol/L)§**	0.2 (0.1, 0.5)	0.4 (0.1, 1.6)	0.5 (0.3, 1.5)*	0.4 (0.2, 0.7)	0.099
**GUDCA (nmol/L)§**	19.5 (10.6, 34.0)	24.0 (15.8, 47.0)	34.6 (19.8, 91.4)*	39.7 (17.1, 60.4)	0.052
**GHCA (nmol/L)§**	2.0 (1.4, 3.2)	1.6 (0.8, 2.5)	1.5 (1.0, 2.2)	2.3 (1.0, 3.0)	0.478
**GCA (nmol/L)§**	15.7 (8.9, 25. 9)	19.2 (13.4, 38.1)	17.2 (5.9, 51.7)	36.8 (22.1, 64.9)*†	0.006
**TUDCA (nmol/L)§**	0.5 (0.4, 0.8)	0.8 (0.5, 1.6)	0.6 (0.5, 2.1)	1.0 (0.6, 2.1)*	0.044
**TaMCA (nmol/L)§**	0.8 (0.4, 1.3)	0.4 (0.2, 0.6)	0.4 (0.2, 0.9)	0.9 (0.4, 1.3)‡	0.041
**THCA (nmol/L)§**	0.4 (0.4, 0.5)	0.4 (0.4, 0.5)	0.4 (0.3, 0.4)	0.4 (0.4, 0.6)	0.382
**TCA (nmol/L)§**	1.3 (0.6, 1.8)	2.3 (1.2, 3.9)	1.0 (0.5, 5.9)	3.5 (2.1, 9.9)*†	0.001
**AlloLCA (nmol/L)§**	0.5 (0.3, 1.0)	0.3 (0.2, 0.3)*†	0.4 (0.3, 0.8)	0.3 (0.3, 0.4)	< 0.001
**isoLCA (nmol/L)§**	2.9 (0.7, 7.9)	0.4 (0.1, 1.3)*†	3.3 (0.3, 6.2)	1.8 (0.2, 5.7)	0.022
**LCA (nmol/L)§**	2.9 (0.9, 6.5)	1.1 (0.1, 3.2)	2.2 (0.5, 6.1)	3.9 (0.8, 6.4)	0.067
**NorDCA (nmol/L)§**	0.9 (0.4, 1.7)	0.3 (0.0, 0.6)*	0.4 (0.2, 1.3)	0.5 (0.3, 1.4)‡	0.007
**7_ketoLCA (nmol/L)§**	4.4 (1. 8, 9.8)	2.2 (0.9, 8.6)	4.5 (3.0, 7.3)	4.5 (2.1, 7.3)	0.368
**12_ketoLCA (nmol/L)§**	2.3 (1.5, 4.6)	0.9 (0.2, 1.7)*†	1.9 (1.1, 5.0)	2.2 (1.3, 5.8)‡	0.006
**CDCA (nmol/L)§**	152.7 (80.0, 342.2)	89.2 (40.0, 289.0)	116.8 (73.2, 197.2)	128.5 (53.4, 371.4)	0.815
**DCA (nmol/L)§**	127.8 (67.7, 226.1)	69.3 (13.9, 114.3)	86.2 (35.4, 142.2)	110.0 (38. 7, 252.2)	0.068
**GLCA (nmol/L)§**	1.4 (0.7, 2.6)	0.7 (0.1, 2.2)†	2.3 (0.6, 5.3)	2.3 (0.8, 8.2)‡	0.032
**LCA_3S (nmol/L)§**	3.8 (1.8, 5.5)	0.6 (0.2, 2.7)*†	2.3 (1.2, 6.2)	2.0 (0.7, 5.2)	0.003
**6_ketoLCA (nmol/L)§**	0.7 (0.6, 0.9)	0.6 (0.4, 0.8)†	0.8 (0.6, 1.3)	0.8 (0.7, 1.0)	0.020
**βCDCA (nmol/L)§**	17.8 (14.8, 20.4)	24.6 (14.6, 38.3)	35.4 (25.1, 71.6)*	29.2 (23.2, 68.3)*	0.004
**βDCA (nmol/L)§**	13.9 (3.4, 22.7)	8.2 (2.0, 14.6)	11.4 (5.7, 22.9)	16.1 (4.7, 29.8)	0.146
**UDCA (nmol/L)§**	27.7 (15.8, 51.2)	26.2 (11.7, 67.7)	43. 2 (18.3, 92.1)	43.2 (17.3, 59.1)	0.603
**HDCA (nmol/L)§**	0.8 (0.2, 1.3)	0.2 (0.0, 0.5)	0.4 (0.1, 0.7)	0.5 (0.2, 1.2)	0.073
**NorCA (nmol/L)§**	1.1 (0.6, 1.7)	1.2 (0.7, 1.8)	1.4 (1.0, 2.2)	2.0 (1.1, 4.5)*	0.028
**7_DHCA (nmol/L)§**	0.7 (0.1, 3.1)	0.7 (0.1, 2.2)	0.9 (0.1, 2. 7)	0.8 (0.2, 1.9)	0.978
**3-DHCA (nmol/L)§**	0.4 (0.2, 1.2)	0.7 (0.3, 1.2)	1.2 (0.3, 1.5)	0.6 (0.2, 1.0)	0.273
**βCA (nmol/L)§**	0.4 (0.2, 0.7)	0.9 (0.6, 2.8)*	1.5 (0.6, 3.8)*	1.2 (0.4, 2.8)*	0.002

**βMCA (nmol/L)§**	1.0 (0.6, 1.4)	0.7 (0.4, 1.0)	0.9 (0.6, 2.4)	0.7 (0.4, 1.2)	0.254
**HCA (nmol/L)§**	7.1 (4.0, 13.0)	4.8 (1.5, 9.6)	5.5 (3.3, 10.5)	4.3 (1.3, 7.0)	0.157
**CA (nmol/L)§**	78.4 (17.4, 349.2)	48.2 (14.6, 169.6)	53.9 (20.1, 104.4)	25.8 (14.8, 150.7)	0.657
**GCDCA (nmol/L)§**	130.3 (51.3, 181.3)	130.3 (74.0, 214.3)	138.9 (87.0, 294.9)	277.0 (110.6, 380.8)*	0.029
**GDCA (nmol/L)§**	29.8 (15.5, 43.6)	15.5 (6.8, 35.8)	25.6 (11.8, 51.0)	60.9 (27.4, 103.7)‡	0.017
**TCDCA (nmol/L)§**	5.6 (2.5, 8.4)	9.1 (4.8, 18.6)	7.3 (2.9, 39.8)	13.5 (6.6, 34.9)*	0.018
**TDCA (nmol/L)§**	2.3 (1.7, 4.1)	1.8 (0.9, 3.3)	2.2 (1.1, 7.0)	4.0 (2.1, 13.2)‡	0.029
**GLCA_3S (nmol/L)§**	57.7 (28.7, 95.7)	16.1 (2.9, 39.2)†	52.5 (13.7, 80.0)	55.5 (27.1, 101.2)‡	0.022
**CDCA_3Gln (nmol/L)§**	4.5 (3.1, 11.4)	4.4 (2.1, 10.9)	5.5 (2.8, 8.7)	5.5 (2.8, 17.1)	0.673
**Valine (μmol/L)**	151.1 ± 24.7	207.1 ± 45.7 *†	178.8 ± 28.4 *	225.4 ± 35.7 *†	< 0.001
**Leucine (μmol/L)**	96.3 ± 15.0	125.7 ± 27.2 *†	109.2 ± 19.6	133.6 ± 22.0 *†	< 0.001
**Isoleucine (μmol/L)**	42.2 ± 7.4	56.7 ± 16.3*†	48.8 ± 9.6	62.8 ± 13.2*†	< 0.001

Data are median (interquartile range). Statistical significance was determined with ANOVA with Bonferroni multiple-comparison analysis. § Log transformed before analysis. * vs NGT-NO, $P < 0.05$; † vs T2D-NO, $P < 0.05$; ‡, vs NGT-O, $P < 0.05$.

NGT-NO, normal glucose tolerance-lean; NGT-O, normal glucose tolerance-abdominally obese; T2D-NO, type 2 diabetes-lean; T2D-O, type 2 diabetes-abdominally obese; βUCA, β-ursocholic acid; UCA, ursocholic acid; GUDCA, glycoursodeoxycholic acid; GHCA, glycohyocholic acid; GCA, glycocholic acid; TUDCA, tauroursodeoxycholic acid; TαMCA, tauro α-muricholic acid; THCA, taurohyocholic acid; TCA, taurocholic acid; alloLCA, allolithocholic acid; isoLCA, isolithocholic acid; LCA, lithocholic acid; NorDCA, 23-nordeoxycholic acid; 7-ketoLCA, 7-ketolithocholic acid; 12-ketoLCA, 12-ketolithocholic acid; CDCA, chenodeoxycholic acid; DCA, deoxycholic acid; GLCA, glycolithocholic acid; LCA-3S, lithocholic acid-3-sulfate; 6-ketoLCA, 6-ketolithocholic acid; βCDCA, 3β-chenodeoxycholic acid; βDCA, 3β-deoxycholic acid; UDCA, ursodeoxycholic acid; HDCA, α-hyodeoxycholic acid; NorCA, norcholic acid; 7-DHCA, 7-ketodeoxycholic acid; 3-DHCA, 3-dehydrocholic acid; βCA, 3β-cholic acid; βMCA, β-muricholic acid; HCA, hyocholic acid; CA, cholic acid; GCDCA, glycochenodeoxycholic acid; GDCA, glycodeoxycholic acid; TCDCA, taurochenodeoxycholic acid; TDCA, taurodeoxycholic acid; GLCA-3S, glycolithocholic acid-3-sulfate; CDCA-3Gln, chenodeoxycholic acid-3-β-d-glucuronide.

Table S4 Bile acids levels of mice (n=31)

Variables (nmol/l)	Control (n=8)	HS-water (n=7)	HS-hk AKK (n=8)	HS-AKK (n=8)	*P*
**TUDCA§**	4.8 (3.5, 5.6)	5.8 (5.4, 12.3)	5.3 (4.4, 6.2)	6 (5.3, 8.4)	0.237
**THDCA§**	4.9 (4.1, 5.8)	5.9 (4.7, 8.3)	3.9 (2.7, 5.9)	5.2 (3.8, 6.4)	0.340
**TωMCA§**	75.5 (58.9, 101.9)	66.5 (35.7, 71)	57.7 (24.6, 87.6)	98.2 (46.4, 146.5)	0.593
**TαMCA§**	42.6 (27.4, 61.6)	12.2 (8.9, 50.6)	16.5 (9.8, 25.2)	35.5 (25, 55.1)	0.230
**TβMCA§**	72.7 (42, 87.1)	53.6 (38.4, 172.3)	90.3 (49.9, 153.8)	192.5 (105.2, 437.9)	0.061
**TCA§**	34.7 (18.5, 40.3)	18.8 (7.8, 44.1)	22.8 (16.8, 33.1)	34 (27.7, 56.8)	0.816
**LCA§**	3.8 (3.4, 5.1)	5.3 (4.2, 6) ‡	3.3 (2.5, 4.3)	2.5 (2.2, 3.1)	0.009
**NorDCA§**	3.9 (2.8, 4.4)	9 (5.1, 12.1)	4.2 (2.7, 5.6)	2.7 (2.4, 3.7)	0.057
**12_ketoLCA§**	3.4 (2.7, 4.4)	4.8 (4.2, 5.8)	4.2 (2.5, 5)	2.7 (2.5, 3.2)	0.074
**CDCA§**	10.5 (5.6, 15.7)	13.1 (6.2, 16.1)	4.7 (1.8, 9.3)	4.2 (2.2, 7)	0.438
**DCA§**	35.5 (25.8, 124.7)	104.2 (65.9, 106.7)	43.2 (16.3, 92.3)	31.1 (24.7, 32.2)	0.082
**UDCA§**	18.9 (12.4, 31.2)	26.2 (10.3, 31.4)	9.2 (5.5, 19.1)	5.7 (4.7, 11.5)	0.095
**HDCA§**	9 (7.5, 14.6)	21.5 (10.9, 24.8) ‡	6 (4.6, 9.3)	4.8 (3.6, 6.2)	0.004
**3_DHCA§**	4.9 (3.9, 5.9)	3.7 (3.5, 4)	3.7 (3.3, 4.3)	3.5 (3.1, 3.7)	0.152
**ωMCA§**	185.7 (113.5, 228.4)	210.2 (147.4, 228.5)	87.2 (68.5, 110.9)	129.4 (100.2, 187.9)	0.048
**αMCA§**	12 (8.3, 15.5)	6.9 (4.6, 9.6)	3.6 (2.8, 7.3)	3.6 (3.2, 6.7)	0.062
**βMCA§**	101.3 (60, 188.6)	120.7 (90.7, 211.4)	88.1 (52.7, 125)	107.1 (93.4, 174.5)	0.669
**ACA§**	12.4 (7, 24.4)	13.2 (7.6, 23.2)	4.3 (2, 8.2)	6.3 (5, 8.5)	0.237
**CA§**	82 (28.7, 111.2)	32.2 (21.3, 64.4)	14.2 (4.3, 32.5)	4.4 (3, 24.3)	0.065
**TCDCA§**	4.3 (3.4, 5.6)	3.3 (2.8, 4.2)	3.4 (2.6, 4)	3.5 (3.1, 4.2)	0.639
**TDCA§**	8.1 (5.8, 16.5)	21 (9.4, 32.8)	9.9 (4.4, 23.6)	5 (4, 10.7)	0.246
**UCA§**	3.5 (2.9, 8.9)	3 (2.8, 3.4)	2.8 (2.6, 4.1)	3 (2.8, 4)	0.161
**6_ketoLCA§**	2.5 (2.2, 2.8)	2.8 (2.7, 3.5)	2.5 (2.2, 3.2)	2 (1.9, 2.4)	0.075
**muroCA§**	2.3 (2.2, 3.8)	6.5 (3.1, 8.2) ‡	2.8 (2.3, 4.3)	1.9 (1.8, 2)	0.054
**βDCA§**	1.9 (0.9, 3.4)	1.9 (1.7, 3)	1.7 (0.1, 3.6)	0.5 (0.3, 1.1)	0.149
**HCA§**	3.5 (2.5, 4.2)	1.3 (1.3, 1.6) †	1.4 (1.3, 1.4) †	1.6 (1.6, 1.8) †	< 0.001
**βCDCA§**	1.3 (0.6, 1.7)	3.6 (2.8, 4.5) †‡	1.8 (0.5, 3.6)	1.1 (0.9, 1.4)	0.028

Data are median (interquartile range). Statistical significance was determined with ANOVA with Bonferroni multiple-comparison analysis. § Log transformed before analysis. † vs control, *P* < 0.05；‡, vs HS-AKK, *P* < 0.05. Mice fed the HS diet were treated with drinking water (HS-water) or water with viable (HS-AKK) or heat-killed A. muciniphila (HS-hk AKK). TUDCA, tauroursodeoxycholic acid; THDCA, taurohyodeoxycholic acid; TωMCA, tauro ω- muricholate; TαMCA, tauro α-muricholate; TβMCA, tauro β-muricholate; TCA, taurocholic acid; LCA, lithocholic acid; NorDCA, 3α,12α-dihydroxynorcholanate\23-nordeoxycholic acid; 12_ketoLCA, 12-ketolithocholic acid; CDCA, chenodeoxycholic acid; DCA, deoxycholic acid; UDCA, ursodeoxycholic acid; HDCA, α-hyodeoxycholic acid; 3_DHCA, 3-dehydrocholic acid; ωMCA, ω-muricholic acid; aMCA, α-muricholic acid; bMCA, β-muricholic acid; ACA, Allocholic acid; CA, cholic acid; TCDCA, taurochenodeoxycholate; TDCA, taurodeoxycholate; UCA, ursocholic acid; 6_ketoLCA, 6-ketolithocholic acid;

muroCA, murocholic acid; βDCA, 3β-deoxycholic acid; HCA, γ- muricholic acid\hyocholic acid; βCDCA, 3β-chenodeoxycholic acid.

Table S5 The sequences of primers used in this study

Gene	Forward	Reverse
**Akkermansia muciniphila**	CAGCACGTGAAGGTGGGGAC	CCTTGCGGTTGGCTTCAGAT
**mPGC1-α**	AGACAAATGTGCTTCCAAAAAGAA	GAAGAGATAAAGTTGTTGGTTTGGC
**mG6Pase**	GTGGCAGTGGTCGGAGACT	ACGGGCGTTGTCCAAAC
**mPEPCK**	CACCATCACCTCCTGGAAGA	GGGTGCAGAATCTCGAGTTG
**mFBP2**	ACCCTGACCCGTTACGTTATG	ACATTCACGCTCCCCGAAATC
**mCEBPα**	TGGACAAGAACAGCAACGAGTAC	GCAGTTGCCCATGGCCTTGAC
**mCEBPβ**	CAAGCTGAGCGACGAGTACA	AGCTGCTCCACCTTCTTCTG
**mCpt1**	CAGAGGATGGACACTGTAAAGG	CGGCACTTCTTGATCAAGCC
**mFoxo1**	ACATTTCGTCCTCGAACCAGCTCA	ATTTCAGACAGACTGGGCAGCGTA
**mPDK4**	GATTGACATCCTGCCTGACC	CATGGAACTCCACCAAATCC
**mGAPDH**	TGATGGGTGTGAACCACGAG	GGGCCATCCACAGTCTTCTG
**mFGF15**	GAGGACCAAAACGAACGAAATT	ACGTCCTTGATGGCAATCG
**mCyclophilin**	GGAGATGGCACAGGAGGAA	GCCCGTAGTGCTTCAGCTT
